# First-in-human intracisternal dosing of RGX-111 in severe MPS I is well tolerated and generates sustained neurodevelopment without HSCT

**DOI:** 10.1016/j.ymthe.2026.04.016

**Published:** 2026-04-10

**Authors:** Raymond Y. Wang, Nina Movsesyan, Shih-Hsin Kan, Tammam Beydoun, Mery Taylor, Richard C. Chang, Dawn Phillips, Jenna Burke, Michelle Gilmor, Yoonjin Cho, Paulo Falabella, Laura Pisani

**Affiliations:** 1 Division of Metabolic Disorders, Children’s Hospital of Orange County Specialists, Orange, CA 92868, USA; 2 Department of Pediatrics, University of California, Irvine, Orange, CA 92868, USA; 3 Research Institute, Children’s Hospital of Orange County, Orange, CA 92868, USA; 4 Orange County Pediatric Radiology Associates, Orange, CA 92868, USA; 5 Department of Pediatric Psychology, Children’s Hospital of Orange County, Orange, CA 92868, USA; 6 REGENXBIO Inc., Rockville, MD 20850, USA

**Keywords:** mucopolysaccharidosis, Hurler syndrome, central nervous system, gene therapy, safety, outcome, cognition, intra-cisterna magna, iduronidase, adeno-associated virus

## Abstract

Mucopolysaccharidosis type I (MPS I), caused by α-L-iduronidase enzyme (IDUA) deficiency, manifests multisystemic symptoms from tissue accumulation of undegraded glycosaminoglycans. Severe MPS I (Hurler syndrome) is associated with developmental delay and loss of neurocognition. Although IDUA enzyme replacement therapy and allogeneic hematopoietic stem cell transplantation (HSCT) are standard therapies for Hurler syndrome, gene therapy represents potential alternative treatment of neurodevelopmental disease. We report more than 5 years of clinical, biochemical, and neurocognitive outcomes following a first-in-human, open-label, single-patient, single administration of intra-cisterna magna (ICM) RGX-111 (AAV9.CB7.hIDUA, 1E−10 vector genomes/g brain mass) in a 20-month-old boy with Hurler syndrome with two older affected siblings deceased from HSCT-related complications. The ICM procedure was safe and well tolerated; treatment-emergent adverse events were mild and self-limiting. Crucially, serial brain imaging has been normal. He has never received an HSCT and continues with weekly IDUA infusions. Neurocognitive testing demonstrates ongoing acquisition of developmental abilities, with cognitive, speech, and motor age equivalents measuring one to two standard deviations below the normative mean. His neurodevelopment is significantly above the natural history of untransplanted Hurler syndrome patients. This single-patient experience of central nervous system-directed gene therapy demonstrates therapeutic potential for severe MPS I and other genetic neurodegenerative diseases.

## Graphical abstract

**Figure:**
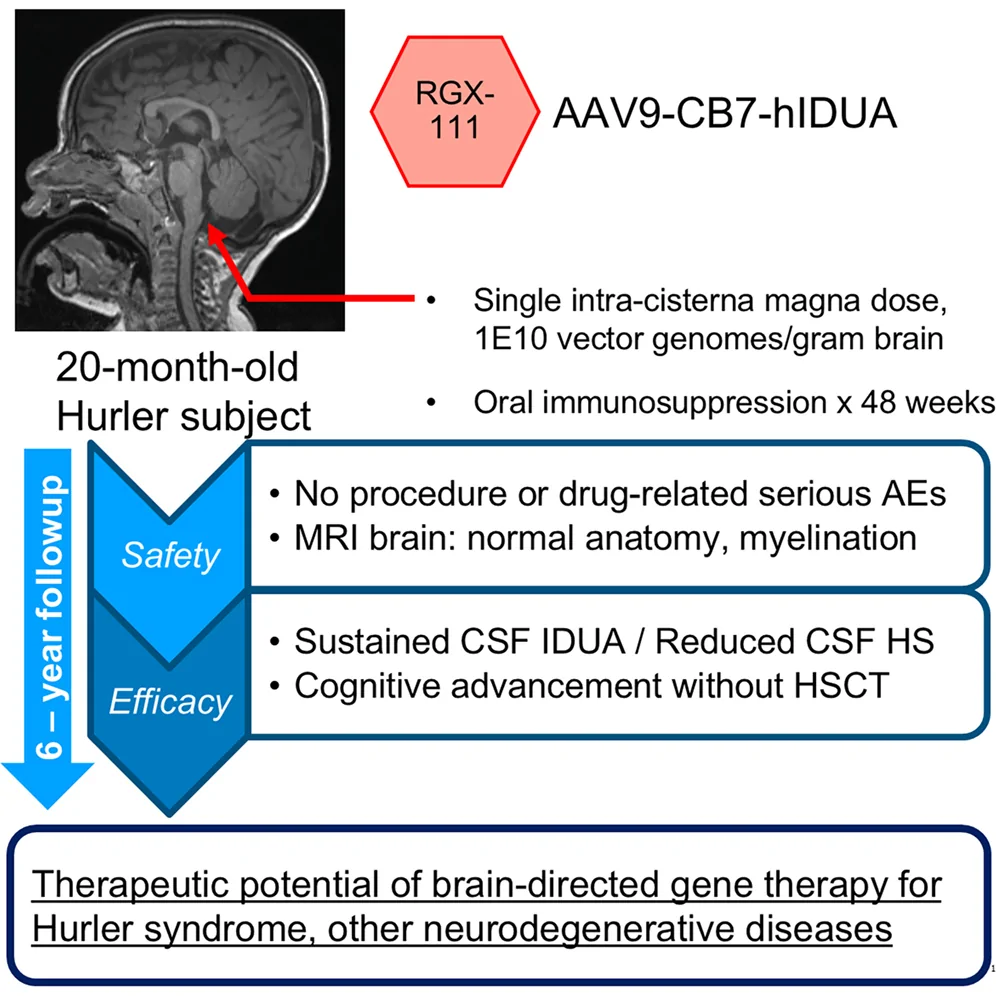

## Teaser

Safety, biochemical, and clinical outcomes of a single-center, single-patient, open-label, first-in-human study of intra-cisterna magna RGX-111 (adeno-associated virus serotype 9 human IDUA) gene therapy in a severe mucopolysaccharidosis type I subject are reported. Six years post dosing, the child, who has never received stem cell transplantation, demonstrates ongoing acquisition of cognitive abilities.

## Introduction

Mucopolysaccharidosis type I (MPS I) is a recessively inherited lysosomal storage disease caused by deficiency of α-L-iduronidase enzyme (IDUA) leading to accumulation of heparan sulfate (HS) and dermatan sulfate (DS) glycosaminoglycans (GAGs) in affected patients’ tissues, including the central nervous system (CNS), with diverse disease sequelae including neurocognitive deficits.^1^^,^^2^ In severe MPS I (Hurler syndrome), CNS HS accumulation is associated with developmental delay and neurocognitive deterioration due to neuroinflammation and hypomyelination.^3^ In untreated Hurler syndrome, symptoms emerge shortly after birth and progress rapidly; most patients exhibit developmental stagnation at 2–3 years of age followed by regression after an initial period of normal development, dying within the first decade of life.^1^ Outside of the CNS, GAG accumulation results in myriad symptoms such as corneal clouding, upper airway obstruction, cardiac valvular dysfunction, hepatosplenomegaly, and skeletal dysplasia.

Intravenous laronidase (recombinant human α-L-iduronidase; Biomarin Pharmaceutical) enzyme replacement therapy (ERT), is one established standard-of-care therapy only for the somatic manifestations of MPS I.^4^ ERT does not cross the blood-brain barrier and therefore cannot treat the neurocognitive sequelae of severe MPS I.^5^ Hematopoietic stem cell transplantation (HSCT) is the standard of care for Hurler syndrome in the United States, demonstrating in multiple cohort studies and meta-analyses improvements in survival, growth, cardiac and respiratory function, mobility, hearing, visual acuity, and CNS manifestations such as neurocognition.^6^ HSCT is able to treat neurocognitive sequelae through donor stem cell migration across the blood-brain barrier, differentiation into CNS microglia, and secretion of IDUA, which the subjects’ own cells internalize. This process enables catabolism of accumulated and continuously produced GAGs in the CNS, resulting in amelioration of neurologic disease. However, risk of death and serious HSCT-related complications such as donor graft failure and chronic graft-versus-host disease still occur, especially with donor-recipient mismatches.^7^

## Results

### Clinical history

The subject was first evaluated at age 2 months. He did not have macrocephaly, frontal bossing, corneal clouding, restricted joint mobility, or organomegaly. Gingival hypertrophy, an umbilical hernia, and large bilateral inguinal hernias were identified. Family history was significant for three older severe MPS I-affected sons; prenatal diagnosis was offered for the subject but declined. The 19-year-old oldest brother, who was nonverbal and had hydrocephalus, severe upper airway obstruction requiring tracheostomy, and severe short stature, did not receive HSCT and was treated with weekly ERT. The two subsequent affected sons underwent HSCT prior to 12 months of age utilizing four of six human leukocyte antigen (HLA) matched umbilical cord blood units but died from complications of severe engraftment syndrome and acute graft-versus-host disease.

Given the family history and examination findings, biochemical and molecular testing were obtained and subsequently confirmed severe MPS I disease. The subject demonstrated markedly elevated urinary total GAGs, elevated urine HS and DS, deficient IDUA enzyme (see Table S1), and the family’s p.R628∗ *IDUA* known pathogenic variant in homozygosity. Diagnostic evaluations identified mild aortic root dilatation (*Z* score +2.5), hepatomegaly (11.3 cm), and dysostosis multiplex.

The child was evaluated for HSCT; an umbilical cord unit with four of six HLA matches was the best identified. HSCT was offered but, given the fatal outcomes in the siblings, the family declined HSCT. The child underwent successful bilateral inguinal herniorrhaphy and central venous catheter placement at 6 months. Intravenous laronidase ERT (0.58 mg/kg/week) was then initiated and has continued to the present day without infusion-associated reactions or adverse events. Prior to dosing with RGX-111 (adeno-associated virus serotype 9 human IDUA [AAV9.CB7.hIDUA]) at 20 months, the subject demonstrated no developmental regression. He was walking unsupported, self-feeding, and spoke three words. Physical examination identified MPS I-related findings, including mild facial coarseness, corneal clouding, lumbosacral gibbus, brachydactyly, and mild contractures of the hip, knee, and elbow joints.

### Clinical outcomes

Baseline brain/spinal magnetic resonance imaging and angiography (MRI/MRA) were obtained to assess the child’s cisterna magna and verify that vascular anatomy was safe for intra-cisterna magna (ICM) access. On the day of treatment, a lumbar puncture was first performed to obtain cerebrospinal fluid (CSF) and inject iopamidol contrast; the subject was placed in Trendelenburg position to enhance visualization of the spinal cord. Following successful computed tomography (CT)-guided access to the cisterna magna with a 25-gauge neonatal spinal needle, 1E−10 vector genome copies/g brain mass (1.265E−13 genome copies) of RGX-111 in 5 mL of Elliotts B artificial CSF was injected over 5 min without adverse effects. The child was then transferred to the pediatric intensive care unit, where he remained sedated for 2 h post procedure to prevent development of CSF leak. He awoke soon after sedation was discontinued, tolerated regular diet on the afternoon of dosing, and was discharged home the day following dosing.

Immunosuppression was initiated 2 days prior to intracisternal injection and consisted of prednisone 0.5 mg/kg/day, followed by a single dose of intravenous methylprednisolone 10 mg/kg administered 60 min prior to RGX-111. After RGX-111 dosing, tacrolimus 0.05 mg/kg twice daily (target trough level, 2–4 ng/mL) and sirolimus 0.25 mg/m^2^/dose twice daily (target trough level, 1–3 ng/mL) were given and adjusted to maintain goal levels. Prednisone, tacrolimus, and sirolimus were well tolerated and safely discontinued at 12, 32, and 48 weeks after RGX-111 dosing.

An echocardiogram performed at baseline identified normal left ventricular systolic function and myocardial thickness. Mitral and aortic valve morphology and function were normal, as was the aortic root diameter (*Z* score = 1.42). At most recent evaluation, all cardiac structure and function measurements continue to be within normal limits. Baseline abdominal ultrasound identified borderline liver and spleen hepatosplenomegaly (10.5 cm and 7.9 cm, respectively), which normalized beginning at week 87 (see Table S2). Serial brain MRI obtained annually post dosing did not identify any neoplasms, dilated perivascular spaces, or white matter abnormalities. Progressive C1-C2 spinal canal stenosis was observed, a finding frequently observed in MPS I patients.

### Adverse events

One serious adverse event, right acute otitis media occurring 5 months after dosing, resulted in a 2-day hospitalization during which the subject’s ear pain and fever resolved following administration of intravenous ceftriaxone. This event was determined to be unrelated to the study treatment and possibly related to immunosuppression. Four nonserious treatment-emergent adverse events occurred in the first 3 weeks of dosing. Of these, three—vomiting, reduced activity level, and reduced appetite—were determined to be possibly related to study treatment and possibly related to immunosuppression. These resolved following administration of one intravenous normal saline bolus. The other adverse event, leukocytosis, resolved without treatment and was determined to be possibly related to study treatment and probably related to prednisone in the immunosuppression regimen. In total, 23 adverse events have been recorded to date (see Table S3); 17 occurred in the first year following dosing, and 16 of those were assessed to be possibly related to immunosuppression. The subject’s most recent adverse events, genu valgum and bilateral knee pain, developed 4 years post dosing, are considered unrelated to RGX-111 or immunosuppression and likely due to somatic MPS I disease progression.

### Biochemical outcomes

**Figure 1:**
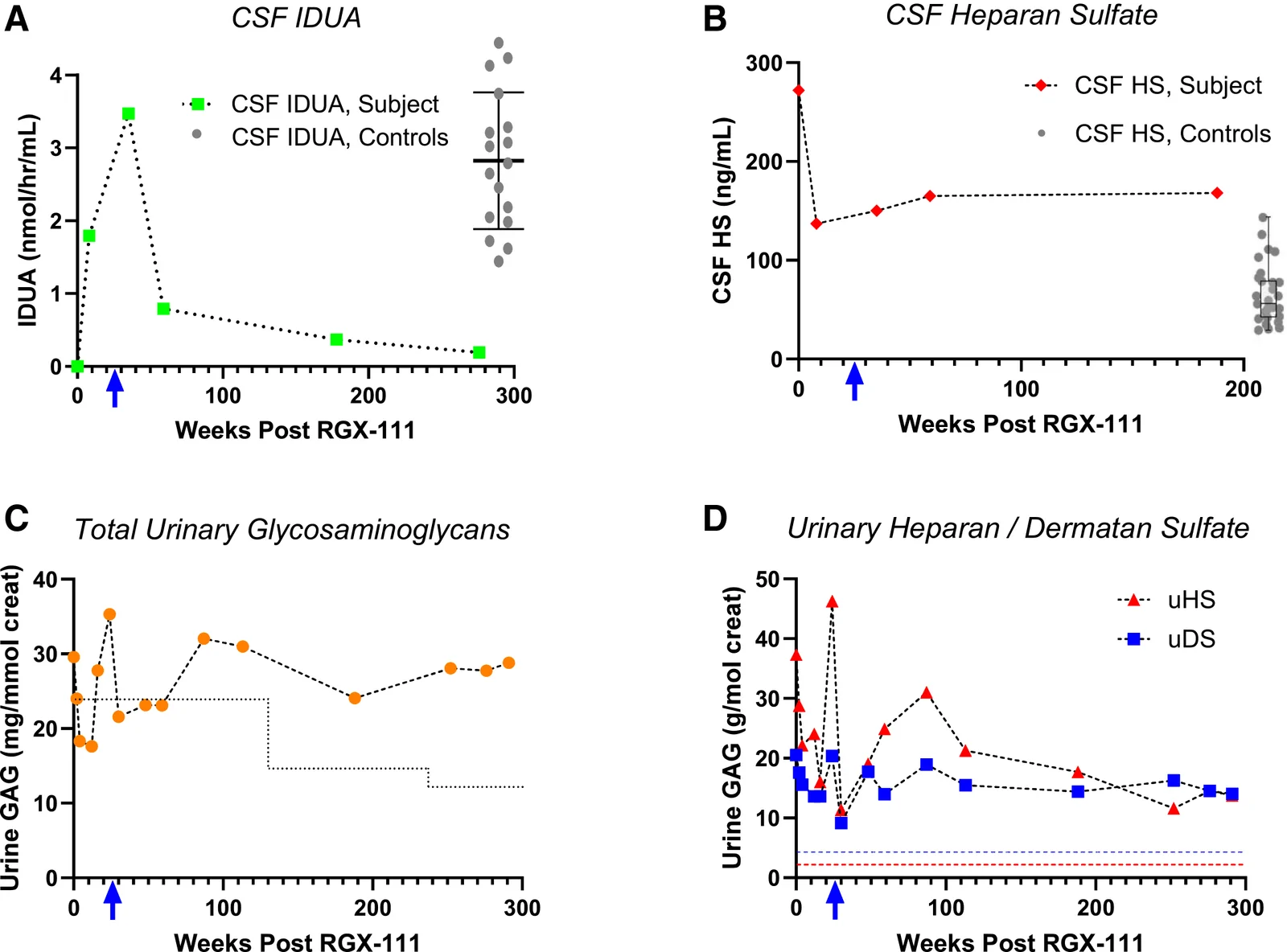
Measurements of CSF IDUA enzyme, CSF heparan sulfate, urine total GAGs, and urine heparan sulfate and dermatan sulfate levels (A) CSF IDUA enzyme levels measured over 290 weeks suggest rapid-onset *IDUA* transgene expression (week 8), which peaked at 35 weeks and subsequently declined after weaning of immunosuppression began (blue arrow). At last measurement, CSF IDUA is 6.8% of mean control CSF IDUA. Gray circles refer to CSF IDUA levels obtained from 17 samples obtained from five individuals without MPS I. (B) CSF heparan sulfate declined rapidly after RGX-111 dosing and remained at or near normal throughout the duration of follow-up. Gray circles refer to CSF HS levels obtained from 29 control individuals without MPS I. (C) Subject’s total urinary GAGs (orange) remained roughly constant throughout the study; though initially at upper limit of normal (dotted black line), it became elevated as the reference range declined with age. (D) Urinary heparan sulfate (uHS) and dermatan sulfate (uDS) levels were essentially unchanged throughout the study. Dotted red and blue horizontal lines indicate the upper limits of normal for uHS and uDS, respectively.

Quantitation of CSF and urinary biochemical data are summarized in Figure 1. The subject’s CSF IDUA activity was below the limit of quantitation at baseline, increased to 1.8 nmol/h/mL (64.7% of mean control CSF IDUA activity) at 8 weeks post dosing, and reached a peak activity of 3.5 nmol/h/mL (125% of mean control IDUA activity) at 35 weeks post dosing. Thereafter, CSF IDUA was measured at 0.79 nmol/h/mL (28.5% of control) IDUA activity at 1 year post dosing, 0.37 nmol/h/mL (13.3% of control) at 188 weeks post dosing, and 0.19 nmol/h/mL (6.8% of control) at 276 weeks post dosing (Figure 1A). The subject’s CSF HS was elevated at baseline (272 ng/mL; mean control CSF HS 69.1 ng/mL, range 140.5–22.0 ng/mL), declined to 137 ng/mL, which was within the normal range (−49.6% from baseline), at week 12, and remained consistently near normal through week 188, the last time point measured to date (Figure 1B). Pre-laronidase plasma IDUA levels were low at baseline and continued to be low throughout the study (data not shown). Urinary total GAGs were slightly elevated at baseline (29.58 mg/mmol creatinine; reference range 24 mg/mmol creatinine) and remained generally constant throughout the study (week 291, 28.82 mg/mmol creatinine, −19.2% from baseline, Figure 1C). Urinary HS (reference range <2.24 g/mol creatinine) and DS (reference range <4.18 g/mol creatinine) were elevated at baseline (37.31 and 20.54 g/mol creatinine, respectively). Although HS and DS were already reduced by more than 90% from diagnosis due to intravenous laronidase ERT, at week 291 both were further reduced to 13.78 and 14.01 g/mol creatinine, respectively (Figure 1D). While CSF IDUA enzyme levels declined after withdrawal of prednisone (week 12) and tacrolimus (week 24), CSF HS levels remained essentially unchanged.

### Body fluid anti-IDUA serologies

**Figure 2:**
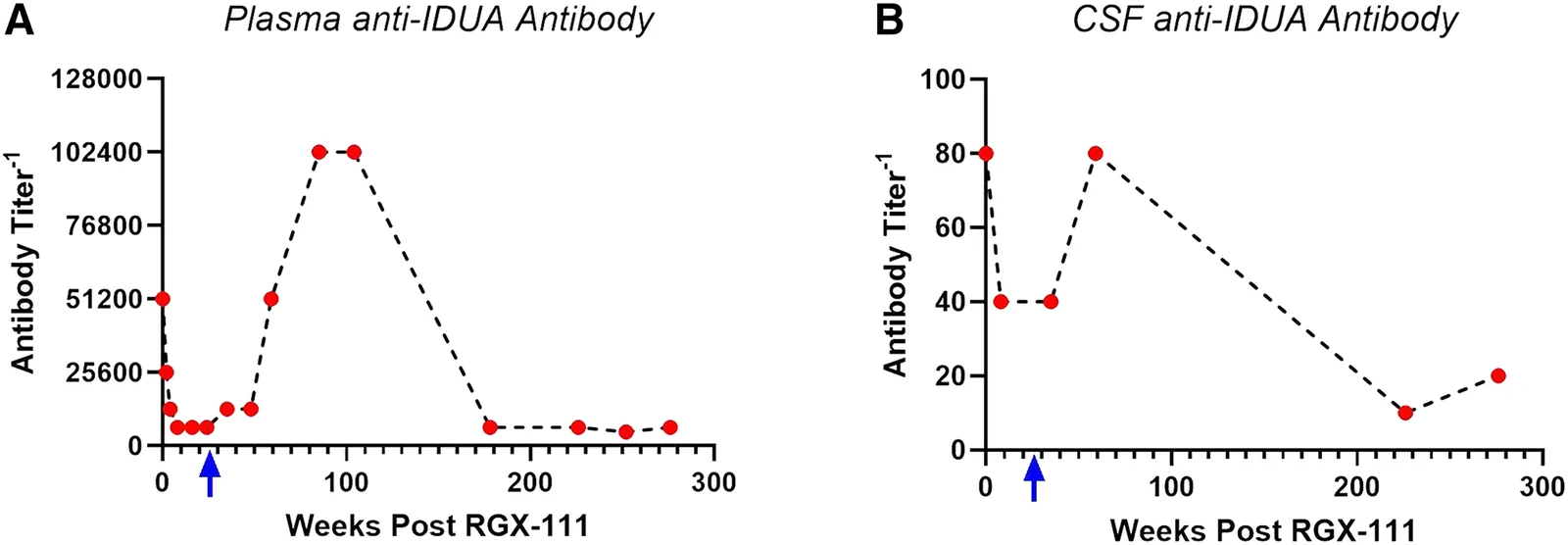
Measurement of plasma and CSF anti-IDUA antibody titers (A) Plasma anti-IDUA antibodies declined with immunosuppression but surged to very high levels between year 1 and year 2 after removal of immunosuppression (blue arrow) before declining to post-RGX-111 levels at years 3, 4, and 5. (B) CSF anti-IDUA antibody titer was 1:80 at baseline and declined following administration of RGX-111 and immunosuppression; after withdrawal of immunosuppression (blue arrow), the titer returned to 1:80 and then decreased at years 4 and 5 to low levels.

Results of plasma and CSF anti-IDUA antibody titers are presented in Figure 2. Plasma anti-IDUA antibody was present at baseline (1:51,200 dilution) and declined to 1:6,400 post dosing and immunosuppression until week 24. As all immunosuppression was withdrawn by week 48, plasma anti-IDUA antibody returned to baseline at week 59 and reached a peak of 1:102,400 dilution at week 85. No infusion-associated reactions were observed with the increase in anti-IDUA titer, although this may have been associated with a slight increase in urinary HS and DS at week 87 that returned to baseline at week 113. Since week 178, the subject has retolerized to laronidase with subsequent antibody levels between 1:4,800 and 1:6,400 (Figure 2A). CSF anti-IDUA was present at baseline (1:80 dilution), declined to 1:40 post dosing and immunosuppression, and returned to 1:80 after removal of immunosuppression. At most recent measurement (week 276), CSF anti-IDUA titer was 1:20 (Figure 2B). Note that despite direct CNS dosing of RGX-111, the ratio of plasma to CSF anti-IDUA titers obtained at the same time ranged from 320:1 to 1,280:1.

### Neurodevelopmental outcomes

**Figure 3:**
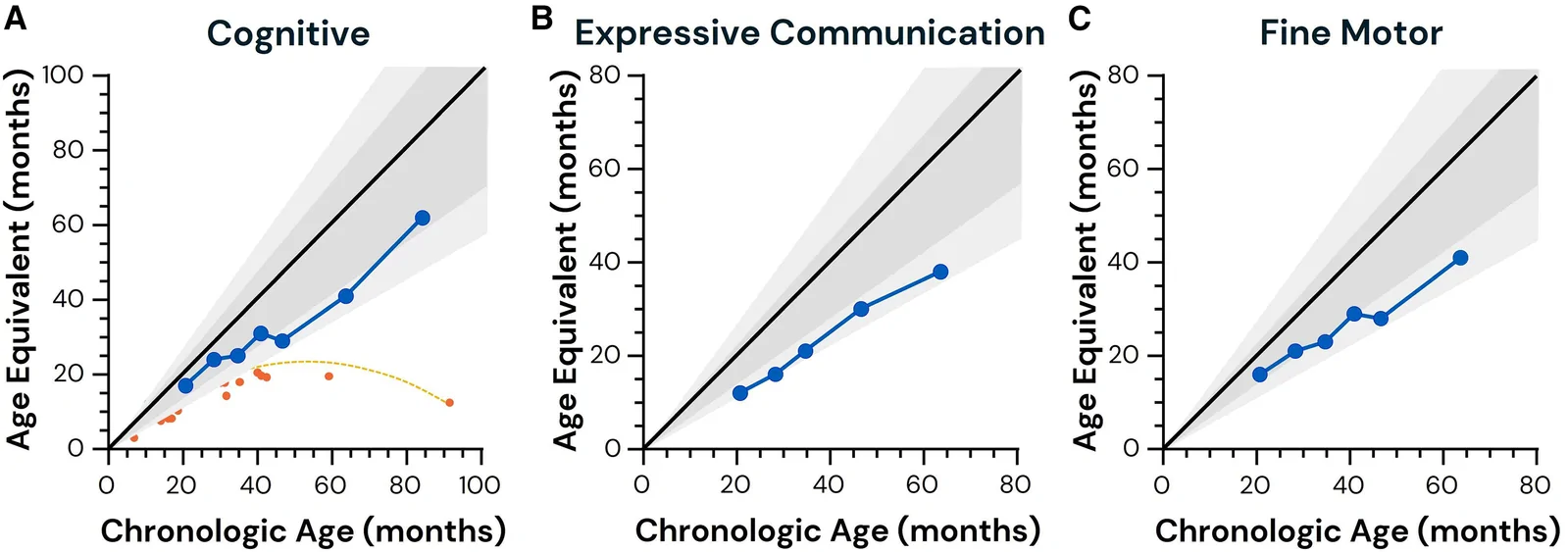
Longitudinal neurodevelopmental age-equivalent scores Longitudinal neurodevelopmental age-equivalent scores (blue circles) for the subject’s (A) cognition, (B) expressive communication, and (C) fine motor function increased with age, indicating ongoing acquisition of developmental milestones without regression. Scores remain between 1 and 2 standard deviations below the mean for age. Data at 72 months chronological age (+52 months post dosing) was not plotted because subject tested above the BSID-III ceiling of 42 months for each category; only cognitive age equivalent is plotted at 84 months chronological age (+64 months post dosing), as the K-ABC does not have a corresponding category for expressive communication and fine motor function. Note that untreated severe MPS I cognitive age equivalents (orange) plateau at an age equivalent of 24 months at a chronological age of 50 months, then progressively decline. Black line indicates the mean (age equivalent = chronological age); the light-gray areas indicate age equivalents ±1 standard deviation, and the dark-gray areas indicate age equivalents ±2 standard deviations from mean.

At baseline, the patient was 20.7 months old with Bayley Scales of Infant and Toddler Development, 3^rd^ Edition (BSID-III) values between 1 and 2 standard deviations below the normative mean, yielding a cognitive age equivalent (AEq) of 17 months (developmental quotient [DQ], 82.1), expressive communication AEq of 12 months (DQ 58.0), and fine motor AEq of 16 months (DQ 77.3). Ongoing skill acquisition at all post-RGX-111 dosing time points was observed. Cognitive, expressive communication, and fine motor AEqs increased at all assessments except for the 26 months post-dosing cognitive AEq, where reduced patient cooperation was documented. The 51-month post-dosing AEqs each exceeded the 42-month AEq BSID-III ceiling, requiring the usage of the Kaufman Assessment Battery for Children (K-ABC) at the 64-month post-dose final assessment, so this point could not be graphed. At a chronological age of 7 years, the subject’s function on the cognitive, expressive communication, and fine motor subtests remained between 1 and 2 standard deviations from the normative mean (Figure 3). Clinically obtained audiology evaluations demonstrate normal hearing. He is bilingual in Arabic and English and is attending primary school in special education first grade with speech therapy (Video S1).

Video S1. Video obtained at subject’s most recent in-person assessment, 5 years 7 months after treatment with RGX-111 Despite never receiving an HSCT, subject is conversant and interactive, fully comprehending the examiner’s questions. He does have coarse facial features and gingival thickening and genu valgum as well as reduced mobility of the fingers and other joints.

## Discussion

This report describes safety and clinical, biochemical, and neurodevelopmental outcomes 5.5 years following the first in-human intracisternal dosing of investigational human *IDUA* gene therapy (RGX-111; REGENXBIO) at 20.7 months of age in a male severe MPS I patient. Although intravenous laronidase was initiated upon diagnosis, its inability to cross the blood-brain barrier necessitated additional therapy to address neurocognitive sequelae of CNS IDUA deficiency and GAG accumulation. The investigational CNS gene therapy was chosen in favor of HSCT due to suboptimal stem cell HLA matching and HSCT-related fatalities in two MPS I-affected older siblings.

While MPS-related skeletal dysplasia increases the challenge of accessing the cisterna magna, a standardized protocol with thorough preprocedural MRI/MRA of the craniocervical junction allows full visualization of the anatomy, including the vertebrobasilar vasculature, to plan optimal strategy for intracisternal access. Our patient tolerated the procedure without complications and was discharged home the day after dosing. Most adverse events occurred in the first year after dosing and were mild. Only three adverse events—reduced activity level, reduced appetite, and vomiting—were considered possibly related to RGX-111 gene therapy, as the symptoms were suggestive of a viral syndrome and took place 1 week after dosing. One serious adverse event, acute otitis media, resulted from hospitalization owing to concern of mastoiditis; however, a CT scan did not identify mastoid fluid, and the child was discharged home with oral amoxicillin/clavulanate. Although no formal nerve conduction studies were performed, due to the protocol being a treatment investigational new drug (IND), the subject did not complain of paresthesias and did not demonstrate abnormal deep tendon reflexes, suggesting there was no clinically significant dorsal root ganglion (DRG) toxicity. This is very important, as toxicity to DRG sensory neurons was first noticed in animal model systems dosed with intravenous AAV^8^; our patient experience is in agreement with the neuropathology of nonhuman primates intracisternally dosed with AAV human *IDUA*, which did not identify any DRG lesions.^9^ Finally, given the recent identification of an intraventricular CNS tumor demonstrating vector genome integration in a 5-year-old severe MPS I patient who had received ICM RGX-111 in the phase 1/2 open-label study (NCT03580083) at age 1 year,^10^ the absence of brain or spine neoplasms in our child more than 5.5 years post RGX-111 is important to note. The child will continue to undergo annual brain and spine MRI surveillance for any CNS abnormality, especially neoplasm.

Rapid biochemical evidence of CNS transduction was evident, with measurable CSF IDUA enzyme activity and nearly 50% reduction in CSF HS observed 8 and 12 weeks, respectively, after treatment. These data also support that CSF GAG measurements may be an early biomarker that is reasonably likely to predict clinical benefit. Moreover, the child’s continued developmental gains in the 5 years after dosing suggests potential efficacy of the investigational intra-CNS *IDUA* gene therapy. The child’s most recent cognitive AEq of 62 months exceeds the highest cognitive AEq achieved (25 months) in a study of 32 untransplanted severe MPS I patients, whose AEq trajectories inflected toward regression at 50 months.^11^ Enrollment in school and initiation of developmental therapies is also expected to continue improving his cognitive function, expressive communication, and motor skills. Although the subject’s most recent CSF enzyme activity declined to 6.8% of control, his CSF HS did not increase appreciably. This is consistent with the abundant clinical experience with allogeneic HSCT for treatment of severe MPS I, where small amounts of CSF IDUA appear sufficient to reduce HS levels and preserve neurocognition.^12^^,^^13^ The biochemical and neurocognitive outcomes of this first-in-human dosing of RGX-111 indicate that intracisternal gene therapy for severe MPS I averts the severe neurodegenerative natural history of the condition without the significant risks of immunocompromise, engraftment syndrome, donor graft failure with autologous recovery, and death presented by the current standard of care, which is allogeneic HSCT.

A limitation of the investigational intracisternal gene therapy in this child is the need to continue intravenous ERT for somatic MPS I disease. We did not observe any appreciable changes in plasma IDUA activity, urinary GAGs, HS, or DS after RGX-111 dosing. This is clinically consistent with the development of bilateral corneal clouding, cervical spinal stenosis, thoracolumbar kyphosis, joint contractures, and genu valgum in the subject; ophthalmologic and orthopedic symptoms of MPS I are well described to persist despite intravenous ERT.^14^^,^^15^ The development of MPS I orthopedic manifestations in the subject is in contrast to the outcomes of eight severe MPS I children treated with lentiviral IDUA autologous hematopoietic stem progenitor cell transplantation (NCT03488394), who expressed supranormal blood IDUA enzyme levels and demonstrated improvement of shoulder and knee joint mobility in conjunction with expression of CSF IDUA, reduction of CSF HS, and progressive acquisition of cognitive skills.^16^^,^^17^^,^^18^^,^^19^

While these data are from a single patient, they suggest that RGX-111 gene therapy delivered into the CNS to treat MPS I generates IDUA expression and GAG clearance within the brain, which are associated with sustained biochemical effects and cognitive benefits, more rapidly than conventional allogeneic HSCT, which requires time for stem cells to engraft, migrate to the brain, and secrete IDUA for cross-correction and catabolism of GAGs. In conjunction with identification of presymptomatic Hurler syndrome infants with universal newborn screening, investigational gene therapy has the potential to allow earlier neurologic benefit as typically HSCT-treated patients experience neurocognitive decline for up to a year before stabilizing and increasing.^2^ Universal newborn screening for MPS I has enabled early oligosymptomatic diagnosis and initiation of CNS-directed therapies to likely further enhance neurocognitive outcomes. HSCT-related adverse effects, including hepatic veno-occlusive disease, thrombotic microangiopathy, donor graft failure with autologous recovery, and death could potentially be avoided. Long-term clinical, biochemical, and neurocognitive follow-up is in progress to document the trajectory of CSF IDUA, HS, neurodevelopment, and other markers in this subject in addition to others who have received intracisternal RGX-111 as participants in the phase 1/2 open-label study.

## Materials and methods

### Human subjects

Following Food and Drug Administration approval of a single-patient IND application for an open-label, single-dose, ICM administration of RGX-111 (#19145), and provision of parental informed consent (see supplemental text: informed consent, CHOC IRB #190671), screening procedures were initiated at 20.3 months chronological age. Parents provided consent for videography and photography as well as sharing of video images for publication purposes. Immunosuppression (for full regimen, see Supplemental Text, Protocol) was continued for a total of 48 weeks after RGX-111 dosing. To summarize, sirolimus 1 mg/m^2^/dose × 3 doses every 4 h was given orally 2 days prior to RGX-111; 0.25 mg/m^2^/dose was given orally twice a day for the next 48 weeks, which achieved the target trough blood levels of 1–3 ng/mL. Methylprednisolone 10 mg/kg was administered one time, intravenously, 30 min prior to ICM RGX-111. Prednisone 0.5 mg/kg/dose was given orally once daily beginning the day after RGX-111 for 2 weeks, reduced to 0.35 mg/kg/dose once daily for 2 weeks, further reduced to 0.2 mg/kg/dose once daily for 2 weeks, and finally 0.1 mg/kg/dose once daily for 2 weeks. Tacrolimus 0.05 mg/kg/dose given orally twice daily was initiated the day after RGX-111 for 24 weeks, which was sufficient to maintain trough blood levels of 2–4 ng/mL, then reduced by 12.5% per week until discontinuation at 32 weeks. Follow-up assessments have been performed periodically for more than 5.5 years after dosing. At each follow-up visit, biomarker laboratory assessments were performed prior to administration of intravenous laronidase ERT.

### Biochemical quantifications

IDUA enzyme activity was assessed using 10 μL of CSF or plasma incubated with 4-methylumbelliferyl α-L-iduronide substrate (final concentration 0.25 mM, Biosynth) in formate buffer (pH 3.5) for 1 h at 37°C in a 96-well plate. Reactions were quenched with 180 μL of glycine carbonate buffer (pH 10.5), and fluorescence measurements were obtained using an Infinite M Plex spectrofluorophotometer (Tecan) at excitation and emission wavelengths of 360 nm and 450 nm, respectively. Bovine serum albumin was utilized as negative control for the IDUA reaction. Control CSF IDUA was measured from 17 pediatric subjects with mitochondrial disorders (CHOC IRB #130990). Measurements were made in triplicate and blinded to subject identity and treatment administered. Substrate background was determined using 10 μL of formate buffer instead of the biological sample; the limit of detection was defined as any signal exceeding 10% of background and was identified to be 0.15 nmol/h/mL.

CSF HS was determined using a proprietary liquid chromatography-tandem mass spectrometry (LC-MS/MS) method developed at CAP-accredited and CLIA-certified Frontage Laboratories (Exton, PA). In brief, HS polysaccharides were digested by heparanases into disaccharides, which were then derivatized before being analyzed with LC-MS/MS; quantitation was performed using calibration curves generated from isotope-labeled internal standard. Control CSF HS was measured in 29 samples obtained from unaffected individuals. Quantification of total urinary GAGs was performed at CAP-accredited and CLIA-certified Greenwood Genetic Center Biochemical Genetics Laboratory (Greenwood, SC) using 1,9-dimethyl-methylene blue incorporation and spectrophotometry, and results were normalized to urine creatinine.

### Neurodevelopmental assessments

Neurodevelopmental outcomes were assessed at baseline and annually post RGX-111 dosing by one rater (M.T.) initially using BSID-III. BSID-III AEq data are presented from the cognitive, expressive communication, and fine motor subtests. The AEq score represents the mean age at which the subtest raw score is obtained in the normative sample. Patient AEq is presented by chronological age in the figures together with standard deviations from the normative mean. Normative data and standard deviations of AEqs were derived from standard scores from the BSID-II manual.^20^ When the subject’s AEq scores exceeded 48 months, the ceiling age for the BSID-III, the K-ABC was utilized.

## Data and code availability

De-identified participant data and images collected for the study will be made available for review upon reasonable request to the corresponding author with a signed data access agreement. The study protocol and informed consent are available as supplemental information.

## Acknowledgments

The investigational RGX-111 product was provided by 10.13039/100013394REGENXBIO. The study was partially supported by REGENXBIO. R.Y.W. and S.-H.K. receive support from the 10.13039/100002001Campbell Foundation of Caring.

## Author contributions

R.Y.W., conceptualization, methodology, supervision, project administration, investigation, data curation, visualization, writing – original draft, and writing – review & editing; N.M., project administration, investigation, data curation, and writing – review & editing; S.-H.K., resources, investigation, validation, visualization, and writing – review & editing; T.B., investigation and writing – review & editing; M.T., investigation and writing – review & editing; R.C.C., investigation and writing – review & editing; D.P., methodology, resources, and writing – review & editing; J.B., methodology, funding acquisition, and writing – review & editing; M.G., methodology, resources, and writing – review & editing; Y.C., methodology and writing – review & editing; P.F., supervision, resources, funding acquisition, and writing – review & editing; L.P., supervision, resources, and writing – review & editing.

## Declaration of interests

D.P., J.B., M.G., Y.C., P.F., and L.P. are employees of REGENXBIO, Inc.

## Supplemental information

Document S1. Supplemental text: Protocol

Document S2. Tables S1–S3

Document S3. Article plus supplemental information
