# Supplementary material for: First-in-human intracisternal dosing of RGX-111 in severe MPS I is well tolerated and generates sustained neurodevelopment without HSCT
Source: Mol Ther. 2026 Apr 10;34(6):3217–23. doi: 10.1016/j.ymthe.2026.04.016 (PMC13239742; doi:10.1016/j.ymthe.2026.04.016)
Supplement: Supplementary file 1 [file mmc1.pdf]

YMTHE, Volume *34*

Supplemental Information

First-in-human intracisternal dosing of RGX-111 in severe MPS I is well tolerated and generates sustained neurodevelopment without HSCT

Raymond Y. Wang, Nina Movsesyan, Shih-Hsin Kan, Tammam Beydoun, Mery Taylor, Richard C. Chang, Dawn Phillips, Jenna Burke, Michelle Gilmor, Yoonjin Cho, Paulo Falabella, and Laura Pisani

Supplemental Text: Protocol

Physician Initiated, Single Center, Single Patient Administration of Intracisternal RGX-111 Gene Therapy for Severe Mucopolysaccharidosis Type I

Protocol Date: 11 July 2019

Sponsor / Principal Investigator: Raymond Wang, M.D.

Sub Investigators: Richard Chang, M.D., Tammam Beydoun, D.O.

Drug: RGX-111 (AAV9.CB7.hIDUA, recombinant adeno-associated virus serotype 9 capsid containing human α-L-iduronidase expression cassette)

Treatment Site: CHOC Children's Hospital, Orange, California

Contact Information:

Name: Raymond Y. Wang, M.D.
Tel: (714) 509-8443
Fax: (714) 509-4531
Email: rawang@choc.org

Study Medication Provider: REGENXBIO, Inc

IND Number: 19145

CHOC IRB Number: 190671

Rationale for Intended Use of Drug
The subject is a 16 month old, 12 kg male child with severe MPS I (*IDUA* homozygous p.R628*/R628*, known pathogenic mutation). Family history: 3 older affected siblings with identical condition. Oldest sibling diagnosed too late for hematopoietic stem cell transplantation (HSCT), which is the standard of care for severe MPS I, and is currently 18 years old with severe disease manifestations including: nonambulatory, nonverbal with severe hydrocephalus, corneal clouding / severely reduced visual acuity, airway obstruction, tracheostomy/gastrostomy dependent.

Subject's second and third oldest siblings underwent HSCT but their non-Caucasian ethnicity made for poor marrow/cord blood stem cell matches and best options were 4 of 6 HLA match umbilical cord lots. Both siblings died from transplant-related complications due to suboptimal matching; subject would suffer a likely outcome of death as recent assessments of available stem cell lots do not offer any better matched candidates.

There are no other standard of care therapies that can preserve neurocognitive function in severe MPS I. Enzyme replacement therapy (ERT) with Aldurazyme® (laronidase, recombinant human alpha iduronidase enzyme, Biomarin Pharmaceuticals, Novato, CA) does not cross the blood brain barrier and cannot treat the neurocognitive regression that occurs in severe MPS I.

Nonclinical studies have shown that RGX-111, an adeno-associated virus (AAV)-mediated gene transfer of IDUA, when delivered into the central nervous system (CNS), can increase IDUA protein expression in a robust and rapid manner, resulting in rapid and near-complete correction of biochemical changes resulting from IDUA deficiency. (Hinderer 2016). Evidence of positive systemic effects were observed as well. Treatment with RGX-111 offers a potential therapeutic approach to address the CNS manifestations of MPS I. Published literature indicates intervention with stem cell transplant must occur ideally prior to 16 months for preservation of neurocognitive function, so for best neurocognitive outcomes this study proposes a one-time, intracisternal (IC) administration of 1e10 genome copies (GC) per gram of brain mass of the drug RGX-111 (AAV9.CB7.hIDUA, recombinant adeno-associated virus serotype 9 capsid containing human α-L-iduronidase expression cassette), as soon as possible, with the possibility that RGX-111 will transduce cells of CNS and result in endogenous synthesis of human iduronidase in the CNS to mitigate neurocognitive deterioration and preserve neurocognitive development.

Subject is not eligible for the current RGX-111 study (NCT03580083) as it mandates subjects be 6 years or older. For this reason, this single patient IND request is being made on behalf of subject.

Description of Severe Mucopolysaccharidosis Type I
Mucopolysaccharidosis type I (MPS I) is a recessively inherited genetic lysosomal storage disease caused by a deficiency of α-L-iduronidase (IDUA) leading to an accumulation of glycosaminoglycans (GAGs) in tissues of affected patients. Since GAGs build up in the CNS and the rest of the body, MPS I patients suffer diverse disease sequelae, including neurocognitive deficits. Severe MPS I (Hurler syndrome) typically involves significant developmental delay and cognitive decline. In untreated Hurler syndrome, symptoms emerge shortly after birth and progress rapidly, such that most patients with Hurler syndrome exhibit severe cognitive decline at 2 – 3 years of age after an initial period of normal development and die within the 1st decade of life.

Hematopoietic stem cell transplantation is the standard of care for Hurler syndrome in the United States, demonstrating in multiple cohort studies and meta-analyses improvements in survival, growth, cardiac and respiratory function, mobility, hearing, visual acuity, and CNS manifestations such as neurocognition. The rationale for treatment is known as 'cross correction,' in which the subject's own cells internalize α-L-iduronidase that is secreted from grafted stem cells which have taken residence within the subject. The internalized enzyme then breaks down the GAGs and mitigates storage-related symptoms.

While intravenous Aldurazyme® ERT is established standard of care for somatic manifestations of MPS I, the drug does not cross the blood-brain barrier and therefore cannot treat the neurocognitive sequelae of severe MPS I. In contrast, HSCT is able to treat neurocognitive sequelae because a subpopulation of donor stem cells

migrates across the blood-brain barrier, differentiates into the immune cells of the CNS, and secretes α-L-iduronidase for the recipient's CNS cells to uptake and cross-correct.

Subject's Medical History and Previous Treatments
Due prior family history, prenatal diagnosis for MPS I was offered to parents, who declined. He was brought for first evaluation at 2 months of age and large bilateral inguinal hernias were identified. Biochemical and molecular testing subsequently confirmed severe MPS I disease, with elevated urinary total GAGs, elevated heparan and dermatan sulfate GAGs, deficient α-L-iduronidase enzyme (1.14 nmol/hr/mg prot, reference range 6 – 71.4), and homozygous *IDUA* p.R628* mutations. Diagnostic evaluations identified mild aortic root dilatation (Z score +2.5), hepatomegaly (11.3 cm), and dysostosis multiplex.

Subject was evaluated for HSCT; the best match was a 4 of 6 umbilical cord unit. HSCT was offered for treatment for subject, but given the fatal outcomes in two prior MPS I affected children – which were due to severe engraftment / graft-versus-host complications – the family declined HSCT.

He began intravenous Aldurazyme® ERT at a standard dose of 0.58 mg/kg/week at 6 months of age and has continued for the past 11 months without infusion-associated reactions or adverse events. He has undergone successful bilateral inguinal herniorrhaphy. Developmentally, he is walking unsupported, is able to feed himself, and has three words. No developmental regression has been noted yet. Objective evaluations at 16 months of age demonstrate normal growth (length 20th percentile, weight 90th percentile, head circumference 94th percentile); mild coarse facial features, minimal corneal clouding, and hip, knee, and elbow joint contractures with a lumbosacral gibbus and brachydactyly. The relative macrocephaly and physical examination findings are consistent with stigmata of MPS I that current Aldurazyme® treatment is unable to fully mitigate. He shall continue on intravenous Aldurazyme® throughout this treatment protocol.

Proposed Method of Administration of Drug, Dose, and Duration of Therapy
Method of administration of RGX-111: computerized tomography-guided intra-cisterna magna injection

Dosage: 1×10^{10} genome copies per gram of patient brain mass (estimated via quantitation of brain magnetic resonance imaging), diluted 1:1 (v/v) with Elliotts B artificial CSF solution.

Duration of therapy: One time administration

Adjunctive (immune suppression) therapy:

Methylprednisolone, 10 mg/kg intravenously, one time prior to RGX-111 administration.

Prednisone, 0.5 mg/kg/day orally, once daily starting day following RGX-111 administration and gradually tapering to discontinuation by +12 weeks following RGX-111 administration.

Tacrolimus, 0.05 mg/kg/dose orally, twice daily starting day following RGX-111 administration with dose titration to achieve target blood level of 2 – 4 ng/mL, x 24 weeks. Dose shall be tapered over 8 weeks to discontinuation by +32 weeks following RGX-111 administration.

Sirolimus, 1 mg/m^2 every 4 hours x 3 doses, 2 days prior to RGX-111 administration, then 0.25 mg/m^2/dose orally, twice daily with dose titration to achieve target blood level of 1 – 3 ng/mL, x 48 weeks.

Prophylactic therapy:

Trimethoprim/sulfamethoxazole, 5 mg/kg orally, Monday / Wednesday / Friday once each dosing day. For prophylaxis against *Pneumocystis carinii* infection

Antifungal prophylaxis against *Candida* and other fungal infections is to be initiated if the ANC is < 500 mm3. The treatment regimen will be determined through local site standard of care.

Description of Clinical Procedures, Laboratory Tests, and Monitoring

Clinical Procedures

Following informed consent provided by parents, baseline assessments would be performed. These assessments can occur up to three weeks prior to RGX-111 dosing to allow for scheduling of all requisite procedures.

Baseline assessments include: medical history and physical examination; height / weight / head circumference; abdominal ultrasound to quantify liver and spleen size; and electrocardiogram.

Neurocognitive testing (Bayley Scales of Infant Development III) will occur at baseline, 24, 48, 78 and 104 weeks.

RGX-111 will be administered via CT guided intra-cisternal injection. The approach shall be from the posterior aspect of the neck, directed towards the foramen of Magendie. CNS anatomy has been reviewed in detail via magnetic resonance imaging. No anatomic anomalies or contraindications to this route have been identified.

Physical and neurologic examination will occur immediately following, and one day after RGX-111 dosing.

Subsequently, these examinations and vital signs will occur at 8, 24, 56, 78, and 104 weeks post-RGX-111 dosing.

Lumbar puncture for assessment of CSF opening pressure and obtaining spinal fluid for laboratory testing will take place at baseline and 8, 24, and 56 weeks after RGX-111 administration.

Subject shall continue to receive weekly, standard-of-care Aldurazyme® ERT throughout this protocol. The option to potentially discontinue intravenous ERT will be based on the clinical judgement of the PI; additional information that may be useful for the decision to stop ERT are trough measurements (based on ERT dosing) of whole blood IDUA activity and urine GAGs up to the Week 24 visit and liver volume by ultrasound. The Week 32, 40, and 48 visits will include additional monitoring of the subject's plasma IDUA activity and urine GAGs levels. IV ERT will be restarted if any of following criteria are met should subject be off Aldurazyme® ERT: increase in urinary GAGs levels 2 times above the level measured at the week 24 visit, or any change in other safety parameters to warrant a restart of IV ERT or as deemed necessary by the PI.

Follow-up abdominal ultrasounds will occur at 24, 56, 78, and 104 weeks post-RGX-111 dosing.
Echocardiogram and electrocardiograms will occur at 56 and 104 weeks post-RGX-111 dosing.
Brain magnetic resonance imaging will be performed at 56 and 104 weeks post-RGX-111 dosing.

Laboratory Tests

Complete blood count (CBC), comprehensive metabolic panel (CMP), lipid panel, coagulation panel, and urinalysis will be obtained before first sirolimus dosing, which is two days prior to administration of RGX-111.

Tacrolimus and sirolimus blood levels for drug titration purposes will be obtained 1, 2, 3, 4, 8, 16, and 24 weeks following RGX-111 administration. Since tacrolimus will be discontinued by week 32, only sirolimus levels will be obtained at 32 and 40 weeks post-RGX-111 administration.

CBC, CMP, and urinalysis will be obtained the day following administration of RGX-111; then 2, 4, 8, 16, 24, 32, 40, 48, 56, 78, and 104 weeks afterwards.

Blood (α-L-iduronidase enzyme activity and GAGs) and urine biomarkers (GAGs) will be obtained at baseline and at 2, 4, 8, 16, 24, 32, 40, 48, 56, 78, and 104 weeks (ideally coinciding with pre-Aldurazyme® ERT for trough α-L-iduronidase quantification) following RGX-111 administration.

Serum and urine RGX-111 quantitative PCR will be obtained at baseline and at 1, 4, 8, 16, and 24 weeks post-RGX-111 dosing.

Serum immune response monitoring (anti-AAV9 and anti-α-L-iduronidase antibody quantification) will be obtained one day prior to, then 2, 8, 16, 24, 32, 56, 78, and 104 weeks post-RGX-111 administration.

ELISPOT assay (for subject's immune response to AAV9 vector and α-L-iduronidase enzyme) will be performed at baseline and at 8, 24, 48, 56, 78, and 104 weeks following RGX-111 administration.

T_{reg} assay will be performed at baseline and at 24, 56, 78, and 104 weeks following RGX-111 administration.

Cerebrospinal fluid testing at baseline, week 8, 24, and 56 will include: protein, glucose, cell count, RGX-111 quantitative PCR, immune response, and biomarker quantification (α-L-iduronidase enzyme activity and GAGs).

Monitoring
Subject will be hospitalized the day prior to, the day of IC RGX-111, and discharged the day after IC RGX-111 if no adverse events are noted (normal activity level, movement, gait, and strength of extremities).

Concomitant medications shall be collected every 4 weeks.

Adverse event / serious adverse event (as defined by CTCAE version 5.0) reporting shall take place throughout the 104 week study period.

Appendix: Information Regarding Devices Utilized to Deliver RGX-111 via IC Injection

Ancillary Components included in the “Pharmacy Kit for Intrathecal / Intracisternal Administration (containing commercial ambient diluent)”

Component	Vendor	Part Number	Quantity Included	Description of Component Use	Regulatory Status
1 mL syringe	Becton Dickinson (BD™)	309628	2	Withdrawal of IP and diluent and prepared dose	K110771[a]
3 mL syringe	Becton Dickinson (BD™)	309657	2		
5 mL syringe	Becton Dickinson (BD™)	309646	2		
10 mL syringe	Becton Dickinson (BD™)	302995	2		
18G Blunt Fill Needle with Filter	Becton Dickinson (BD™)	305211	1	Withdrawal of Elliotts B® Solution	NA[b]
18G Blunt Fill Needle	Becton Dickinson (BD™)	305180	2	Transfer of withdrawn IP and diluent into dosing syringe	
Alcohol Prep Pads	Uline	S-16183	10	IP vial	NA
Vial Adapters	West Pharmaceuticals	8070117	10	Withdrawal of IP from vials	K001293 [a]
Elliotts B® Solution	Lukare Medical	28002	1	Diluent	NDC<br>55792-007-10
Tip Cap	B Braun	R2000	1	Closure of primed dosing syringe	NDC/WSN<br>08021-6544-96
Sterile Label / Pen	Various	Various	1	Labeling of syringes	NA
Sterility Maintenance Bag	Various	3312ST	1	Storage of prepared dosing syringe	NA

[a]510(k) number

[b]FDA Class 1 exempt since classified as a manual surgical instrument for general use, meets ISO guidelines for needles (7864 and 9626)

Ancillary Components included in the "Surgery Kit for Intrathecal / Intracisternal Administration"

Component	Vendor	Part Number	Quantity Included	Description of Component Use	Regulatory Status
25 G × 2" Short Spinal Needle with Quincke Bevel	Becton Dickinson (BD™)	405078	2	Administration of IP into patient	D018795[a]
25 G × 3" Short Spinal Needle with Quincke Bevel	Becton Dickinson (BD™)	405170	2		
25 G × 4 11/16" Spinal Needle with Quincke Bevel	Becton Dickinson (BD™)	405234	2		
22 G x 5" Spinal Needle with Quincke Bevel	Becton Dickinson (BD™)	405148	2		
20 G x 3" Spinal Needle with Quincke Bevel	Becton Dickinson (BD™)	405172	1	Optional introducer needle for stabilizing 25G spinal needle	
18 G x 3" Spinal Needle with Quincke Bevel	Becton Dickinson (BD™)	405174	1	Optional introducer needle for stabilizing 22G spinal needle	
T-connector extension set with female luer lock adapter, approximate length 6.7"	Baxter	2N3339	1	Administration of IP into patient	K921899[b]
