# Supplementary material for: First-in-human intracisternal dosing of RGX-111 in severe MPS I is well tolerated and generates sustained neurodevelopment without HSCT
Source: Mol Ther. 2026 Apr 10;34(6):3217–23. doi: 10.1016/j.ymthe.2026.04.016 (PMC13239742; doi:10.1016/j.ymthe.2026.04.016)
Supplement: Supplementary file 2 [file mmc2.pdf]

**Informed Consent:** A copy of the most recent IRB-approved consent form is provided separately.

**Supplemental Protocol** has been provided as a separate document.

**Table S1.** Characteristics of the subject at diagnosis and at most recent assessment. Note that urinary GAG reference values decline with increasing age.

| *Parameter* | *Value at Diagnosis (6 months)* | *Most Recent Assessment (78 months of age)* |
|---|---|---|
| Leukocyte α-L-iduronidase (ref 6 – 71.4 nmol/hr/mg) | 1.14 nmol/hr/mg | 1.44 nmol/hr/mg |
| Total urine GAGs | 128.06 mg/mmol creatinine (ref < 53 mg/mmol creatinine) | 28.82 mg/mmol creatinine (ref < 12 mg/mmol creatinine) |
| Urine dermatan sulfate | 161.03 g/mol creatinine (ref < 18.47 g/mol creatinine) | 14.01 g/mol creatinine (ref < 5.06 g/mol creatinine) |
| Urine heparan sulfate | 266.41 g/mol creatinine (ref < 5.28 g/mol creatinine) | 13.78 g/mol creatinine (ref < 1.47 g/mol creatinine) |
| Length / Height | 61.4 cm (52$^{nd}$ percentile) | 107 cm (1$^{st}$ percentile) |
| Weight | 7.14 kg (84$^{th}$ percentile) | 24.9 kg (55$^{th}$ percentile) |
| Occipito-frontal circumference | 45.5 cm (77$^{th}$ percentile) | 51.5 cm (25$^{th}$ percentile) |

**Table S2.** Liver and spleen measurements of the subject, who began treatment with intravenous ERT at age 6 months. Hepatosplenomegaly (**bold**) was present from 14 to 34 months of age; liver and spleen dimensions normalized beginning at Week 87 (41 months of age).

| *Age* | *Liver craniocaudal dimension (cm)* | *Spleen craniocaudal dimension (cm)* |
|---|---|---|
| Week -30 (age 14 months) | **11.3** | **7.6** |
| Baseline (age 21 months) | **10.5** | **7.9** |
| Week 30 (age 28 months) | **11.3** | **8** |
| Week 59 (age 34 months) | **11.2** | **8.1** |
| Week 87 (age 41 months) | 11.2 | 7.7 |
| Week 112 (age 47 months) | 11.2 | 7.8 |
| Week 138 (age 53 months) | 11.1 | 8.7 |
| Week 250 (age 78 months) | 11.2 | 8.7 |

**Table S3.** Listing of adverse events with complete details including causality assessments and outcome.

| Reported Term | Start Date (day) | End Date (day) | Serious | Severity | Relation. to RGX-111 | Related to Proc. Admin. | Relation. to Other Study Proc. | Relation. to IS | Actions Taken | Outcome |
|---|---|---|---|---|---|---|---|---|---|---|
| *Reduced Activity Level* | 2019-10-21 (7) | 2019-10-25 (11) | No | Mild | Possibly Related | No | Not Applicable | Possibly Related | No Dose Change To Immunosuppression. No Study Discontinuation | Recovered/ Resolved |
| *Reduced Appetite* | 2019-10-21 (7) | 2019-10-25 (11) | No | Mild | Possibly Related | No | Not Applicable | Possibly Related | No Dose Change To Immunosuppression. No Study Discontinuation | Recovered/ Resolved |
| *Vomiting* | 2019-10-21 (7) | 2019-10-24 (10) | No | Mild | Possibly Related | No | Not Applicable | Possibly Related | No Dose Change To Immunosuppression. No Study Discontinuation | Recovered/ Resolved |
| *Leukocytosis* | 2019-10-28 (14) | 2019-11-11 (28) | No | Mild | Possibly Related | No | Not Applicable | Possibly Related (Prednisone) | No Dose Change To Immunosuppression. No Study Discontinuation | Recovered/ Resolved |
| *Cough* | 2019-12-01 (48) | 2019-12-05 (52) | No | Mild | Not Related | No | Not Applicable | Not Related | No Dose Change To Immunosuppression. No Study Discontinuation | Recovered/ Resolved |
| *Rhinorrhea* | 2019-12-01 (48) | 2019-12-05 (52) | No | Mild | Not Related | No | Not Related | Possibly Related | No Dose Change To Immunosuppression. No Study Discontinuation | Recovered/ Resolved |
| *Fever* | 2020-01-30 (108) | 2020-02-03 (112) | No | Mild | Not Related | No | Not Applicable | Possibly Related | No Dose Change To Immunosuppression. No Study Discontinuation | Recovered/ Resolved |
| *Rhinorrhea* | 2020-01-30 (108) | 2020-02-05 (114) | No | Mild | Not Related | No | Not Applicable | Possibly Related | No Dose Change To Immunosuppression. No Study Discontinuation | Recovered/ Resolved |

| Reported Term | Start Date (day) | End Date (day) | Serious | Severity | Relation. to RGX-111 | Related to Proc. Admin. | Relation. to Other Study Proc. | Relation. to IS | Actions Taken | Outcome |
|---|---|---|---|---|---|---|---|---|---|---|
| *Alkaline Phosphatase Increased* | 2020-02-07 (116) | 2020-05-12 (211) | No | Moderate | Not Related | No | Not Applicable | Possibly Related | No Dose Change To Immunosuppression. No Study Discontinuation | Recovered/ Resolved |
| *Neutropenia* | 2020-02-07 (116) | 2020-03-01 (139) | No | Moderate | Not Related | No | Not Applicable | Possibly Related | No Dose Change To Immunosuppression. No Study Discontinuation | Recovered/ Resolved |
| *Fever* | 2020-02-28 (137) | 2020-03-01 (139) | No | Mild | Not Related | No | Not Applicable | Possibly Related | No Dose Change To Immunosuppression. No Study Discontinuation | Recovered/ Resolved |
| *Dehydration* | 2020-03-01 (139) | 2020-03-02 (140) | No | Mild | Not Related | No | Not Applicable | Possibly Related | No Dose Change To Immunosuppression. No Study Discontinuation | Recovered/ Resolved |
| *Right Acute Otitis Media* | 2020-03-22 (160) | 2020-03-24 (162) | Yes | Severe | Not Related | No | Not Applicable | Possibly Related | No Dose Change To Immunosuppression. No Study Discontinuation | Recovered/ Resolved |
| *Fever* | 2020-07-28 (288) | 2020-08-01 (292) | No | Mild | Not Related | No | Not Applicable | Possibly Related | No Dose Change To Immunosuppression. No Study Discontinuation | Recovered/ Resolved |
| *Decreased appetite* | 2020-08-02 (293) | 2020-08-04 (295) | No | Mild | Not Related | No | Not Applicable | Possibly Related | No Dose Change To Immunosuppression. No Study Discontinuation | Recovered/ Resolved |
| *Rash* | 2020-08-08 (299) | 2020-08-10 (301) | No | Mild | Not Related | No | Not Applicable | Possibly Related | No Dose Change To Immunosuppression. No Study Discontinuation | Recovered/ Resolved |
| *Fever* | 2020-08-09 (300) | 2020-08-09 (300) | No | Mild | Not Related | No | Not Applicable | Possibly Related | No Dose Change To Immunosuppression. No Study Discontinuation | Recovered/ Resolved |

| Reported Term | Start Date (day) | End Date (day) | Serious | Severity | Relation. to RGX-111 | Related to Proc. Admin. | Relation. to Other Study Proc. | Relation. to IS | Actions Taken | Outcome |
|---|---|---|---|---|---|---|---|---|---|---|
| *Neutropenia* | 2020-11-30 (413) | 2021-06-15 (610) | No | Moderate | Not Related | No | Not Applicable | Not Related | No Study Discontinuation | Recovered/ Resolved |
| *Rhinorrhea* | 2021-06-13 (608) | 2021-06-16 (611) | No | Mild | Not Related | No | Not Applicable | Not Related | No Study Discontinuation | Recovered/ Resolved |
| *Otitis Media* | 2022-09-28 (1080) | 2022-09-28 (1080) | No | Mild | Not Related | No | Not Applicable | Not Related | No Study Discontinuation | Recovered/ Resolved |
| *Otitis Media* | 2023-05-15 (1309) | 2023-05-21 (1315) | No | Mild | Not Related | No | Not Applicable | Not Related | No Study Discontinuation | Recovered/ Resolved |
| *Genu Valgum* | 2023-09-23 (1439) | Ongoing | No | Mild | Not Related | No | Not Related | Not Applicable | Orthopedics Referral | Ongoing |
| *Bilateral Knee Pain* | 2024-01-02 (1547) | Ongoing | No | Mild | Not Related | No | Not Related | Not Applicable | Orthopedics Referral | Ongoing |

**Video S1**: Video was obtained at subject's most recent in-person assessment, five years and seven months after treatment with RGX-111. Despite never receiving a HSCT, subject is conversant and interactive, fully comprehending examiner's questions. He does have coarse facial features and gingival thickening, genu valgum, as well as reduced mobility of the fingers and other joints.
