# Supplementary material for: First-in-human intracisternal dosing of RGX-111 in severe MPS I is well tolerated and generates sustained neurodevelopment without HSCT
Source: Mol Ther. 2026 Apr 10;34(6):3217–23. doi: 10.1016/j.ymthe.2026.04.016 (PMC13239742; doi:10.1016/j.ymthe.2026.04.016)
Supplement: Supplementary file 3 [file mmc4.pdf]

Molecular Therapy
Brief report

First-in-human intracisternal dosing of RGX-111 in severe MPS I is well tolerated and generates sustained neurodevelopment without HSCT

Raymond Y. Wang,[1,2] Nina Movsesyan,[3] Shih-Hsin Kan,[3] Tammam Beydoun,[4] Mery Taylor,[5] Richard C. Chang,[1] Dawn Phillips,[6] Jenna Burke,[6] Michelle Gilmor,[6] Yoonjin Cho,[6] Paulo Falabella,[6] and Laura Pisani[6]

[1]Division of Metabolic Disorders, Children's Hospital of Orange County Specialists, Orange, CA 92868, USA; [2]Department of Pediatrics, University of California, Irvine, Orange, CA 92868, USA; [3]Research Institute, Children's Hospital of Orange County, Orange, CA 92868, USA; [4]Orange County Pediatric Radiology Associates, Orange, CA 92868, USA; [5]Department of Pediatric Psychology, Children's Hospital of Orange County, Orange, CA 92868, USA; [6]REGENXBIO Inc., Rockville, MD 20850, USA

Mucopolysaccharidosis type I (MPS I), caused by α-L-iduronidase enzyme (IDUA) deficiency, manifests multisystemic symptoms from tissue accumulation of undegraded glycosaminoglycans. Severe MPS I (Hurler syndrome) is associated with developmental delay and loss of neurocognition. Although IDUA enzyme replacement therapy and allogeneic hematopoietic stem cell transplantation (HSCT) are standard therapies for Hurler syndrome, gene therapy represents potential alternative treatment of neurodevelopmental disease. We report more than 5 years of clinical, biochemical, and neurocognitive outcomes following a first-in-human, open-label, single-patient, single administration of intra-cisterna magna (ICM) RGX-111 (AAV9.CB7.hIDUA, 1E−10 vector genomes/g brain mass) in a 20-month-old boy with Hurler syndrome with two older affected siblings deceased from HSCT-related complications. The ICM procedure was safe and well tolerated; treatment-emergent adverse events were mild and self-limiting. Crucially, serial brain imaging has been normal. He has never received an HSCT and continues with weekly IDUA infusions. Neurocognitive testing demonstrates ongoing acquisition of developmental abilities, with cognitive, speech, and motor age equivalents measuring one to two standard deviations below the normative mean. His neurodevelopment is significantly above the natural history of untransplanted Hurler syndrome patients. This single-patient experience of central nervous system-directed gene therapy demonstrates therapeutic potential for severe MPS I and other genetic neurodegenerative diseases.

INTRODUCTION

Mucopolysaccharidosis type I (MPS I) is a recessively inherited lysosomal storage disease caused by deficiency of α-L-iduronidase enzyme (IDUA) leading to accumulation of heparan sulfate (HS) and dermatan sulfate (DS) glycosaminoglycans (GAGs) in affected patients' tissues, including the central nervous system (CNS), with diverse disease sequelae including neurocognitive deficits.[1,2] In severe MPS I (Hurler syndrome), CNS HS accumulation is associated with developmental delay and neurocognitive deterioration due to neuroinflammation and hypomyelination.[3] In untreated Hurler syndrome, symptoms emerge shortly after birth and progress rapidly; most patients exhibit developmental stagnation at 2–3 years of age followed by regression after an initial period of normal development, dying within the first decade of life.[1] Outside of the CNS, GAG accumulation results in myriad symptoms such as corneal clouding, upper airway obstruction, cardiac valvular dysfunction, hepatosplenomegaly, and skeletal dysplasia.

Intravenous laronidase (recombinant human α-L-iduronidase; Biomarin Pharmaceutical) enzyme replacement therapy (ERT), is one established standard-of-care therapy only for the somatic manifestations of MPS I.[4] ERT does not cross the blood-brain barrier and therefore cannot treat the neurocognitive sequelae of severe MPS I.[5] Hematopoietic stem cell transplantation (HSCT) is the standard of care for Hurler syndrome in the United States, demonstrating in multiple cohort studies and meta-analyses improvements in survival, growth, cardiac and respiratory function, mobility, hearing, visual acuity, and CNS manifestations such as neurocognition.[6] HSCT is able to treat neurocognitive sequelae through donor stem cell migration across the blood-brain barrier, differentiation into CNS microglia, and secretion of IDUA, which the subjects' own cells internalize. This process enables catabolism of accumulated and continuously produced GAGs in the CNS, resulting in amelioration of neurologic disease. However, risk of death and serious HSCT-related complications such as donor graft failure and chronic graft-versus-host disease still occur, especially with donor-recipient mismatches.[7]

RESULTS

Clinical history

The subject was first evaluated at age 2 months. He did not have macrocephaly, frontal bossing, corneal clouding, restricted joint

Received 10 February 2026; accepted 8 April 2026;
https://doi.org/10.1016/j.ymthe.2026.04.016.

Correspondence: Raymond Y. Wang, Division of Metabolic Disorders, 1201 W. La Veta Ave., Orange, CA 92868, USA.
E-mail: rawang@choc.org

Published by Elsevier Inc. on behalf of The American Society of Gene and Cell Therapy.
This is an open access article under the CC BY license (http://creativecommons.org/licenses/by/4.0/).

mobility, or organomegaly. Gingival hypertrophy, an umbilical hernia, and large bilateral inguinal hernias were identified. Family history was significant for three older severe MPS I-affected sons; prenatal diagnosis was offered for the subject but declined. The 19-year-old oldest brother, who was nonverbal and had hydrocephalus, severe upper airway obstruction requiring tracheostomy, and severe short stature, did not receive HSCT and was treated with weekly ERT. The two subsequent affected sons underwent HSCT prior to 12 months of age utilizing four of six human leukocyte antigen (HLA) matched umbilical cord blood units but died from complications of severe engraftment syndrome and acute graft-versus-host disease.

Given the family history and examination findings, biochemical and molecular testing were obtained and subsequently confirmed severe MPS I disease. The subject demonstrated markedly elevated urinary total GAGs, elevated urine HS and DS, deficient IDUA enzyme (see Table S1), and the family's p.R628* *IDUA* known pathogenic variant in homozygosity. Diagnostic evaluations identified mild aortic root dilatation (Z score +2.5), hepatomegaly (11.3 cm), and dysostosis multiplex.

The child was evaluated for HSCT; an umbilical cord unit with four of six HLA matches was the best identified. HSCT was offered but, given the fatal outcomes in the siblings, the family declined HSCT. The child underwent successful bilateral inguinal herniorrhaphy and central venous catheter placement at 6 months. Intravenous laronidase ERT (0.58 mg/kg/week) was then initiated and has continued to the present day without infusion-associated reactions or adverse events. Prior to dosing with RGX-111 (adeno-associated virus serotype 9 human IDUA [AAV9.CB7.hIDUA]) at 20 months, the subject demonstrated no developmental regression. He was walking unsupported, self-feeding, and spoke three words. Physical examination identified MPS I-related findings, including mild facial coarseness, corneal clouding, lumbosacral gibbus, brachydactyly, and mild contractures of the hip, knee, and elbow joints.

Clinical outcomes

Baseline brain/spinal magnetic resonance imaging and angiography (MRI/MRA) were obtained to assess the child's cisterna magna and verify that vascular anatomy was safe for intra-cisterna magna (ICM) access. On the day of treatment, a lumbar puncture was first performed to obtain cerebrospinal fluid (CSF) and inject iopamidol contrast; the subject was placed in Trendelenburg position to enhance visualization of the spinal cord. Following successful computed tomography (CT)-guided access to the cisterna magna with a 25-gauge neonatal spinal needle, 1E−10 vector genome copies/g brain mass (1.265E−13 genome copies) of RGX-111 in 5 mL of Elliotts B artificial CSF was injected over 5 min without adverse effects. The child was then transferred to the pediatric intensive care unit, where he remained sedated for 2 h post procedure to prevent development of CSF leak. He awoke soon after sedation was discontinued, tolerated regular diet on the afternoon of dosing, and was discharged home the day following dosing.

Immunosuppression was initiated 2 days prior to intracisternal injection and consisted of prednisone 0.5 mg/kg/day, followed by a single dose of intravenous methylprednisolone 10 mg/kg administered 60 min prior to RGX-111. After RGX-111 dosing, tacrolimus 0.05 mg/kg twice daily (target trough level, 2–4 ng/mL) and sirolimus 0.25 mg/m^2/dose twice daily (target trough level, 1–3 ng/mL) were given and adjusted to maintain goal levels. Prednisone, tacrolimus, and sirolimus were well tolerated and safely discontinued at 12, 32, and 48 weeks after RGX-111 dosing.

An echocardiogram performed at baseline identified normal left ventricular systolic function and myocardial thickness. Mitral and aortic valve morphology and function were normal, as was the aortic root diameter (Z score = 1.42). At most recent evaluation, all cardiac structure and function measurements continue to be within normal limits. Baseline abdominal ultrasound identified borderline liver and spleen hepatosplenomegaly (10.5 cm and 7.9 cm, respectively), which normalized beginning at week 87 (see Table S2). Serial brain MRI obtained annually post dosing did not identify any neoplasms, dilated perivascular spaces, or white matter abnormalities. Progressive C1-C2 spinal canal stenosis was observed, a finding frequently observed in MPS I patients.

Adverse events

One serious adverse event, right acute otitis media occurring 5 months after dosing, resulted in a 2-day hospitalization during which the subject's ear pain and fever resolved following administration of intravenous ceftriaxone. This event was determined to be unrelated to the study treatment and possibly related to immunosuppression. Four nonserious treatment-emergent adverse events occurred in the first 3 weeks of dosing. Of these, three—vomiting, reduced activity level, and reduced appetite—were determined to be possibly related to study treatment and possibly related to immunosuppression. These resolved following administration of one intravenous normal saline bolus. The other adverse event, leukocytosis, resolved without treatment and was determined to be possibly related to study treatment and probably related to prednisone in the immunosuppression regimen. In total, 23 adverse events have been recorded to date (see Table S3); 17 occurred in the first year following dosing, and 16 of those were assessed to be possibly related to immunosuppression. The subject's most recent adverse events, genu valgum and bilateral knee pain, developed 4 years post dosing, are considered unrelated to RGX-111 or immunosuppression and likely due to somatic MPS I disease progression.

Biochemical outcomes

Quantitation of CSF and urinary biochemical data are summarized in Figure 1. The subject's CSF IDUA activity was below the limit of quantitation at baseline, increased to 1.8 nmol/h/mL (64.7% of mean control CSF IDUA activity) at 8 weeks post dosing, and reached a peak activity of 3.5 nmol/h/mL (125% of mean control IDUA activity) at 35 weeks post dosing. Thereafter, CSF IDUA was measured at 0.79 nmol/h/mL (28.5% of control) IDUA activity at 1 year post dosing, 0.37 nmol/h/mL (13.3% of control) at 188 weeks

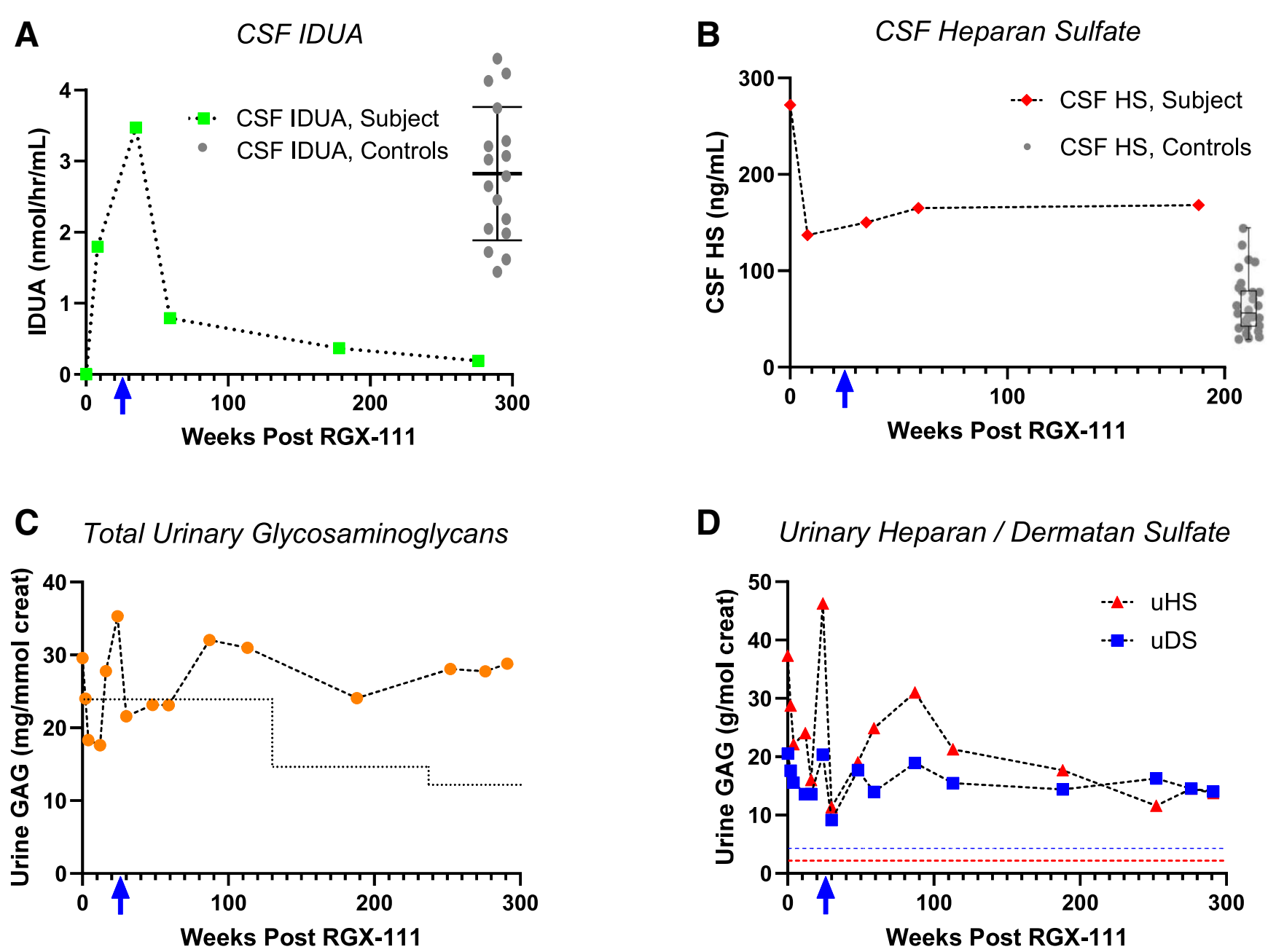

Figure 1. Measurements of CSF IDUA enzyme, CSF heparan sulfate, urine total GAGs, and urine heparan sulfate and dermatan sulfate levels

(A) CSF IDUA enzyme levels measured over 290 weeks suggest rapid-onset *IDUA* transgene expression (week 8), which peaked at 35 weeks and subsequently declined after weaning of immunosuppression began (blue arrow). At last measurement, CSF IDUA is 6.8% of mean control CSF IDUA. Gray circles refer to CSF IDUA levels obtained from 17 samples obtained from five individuals without MPS I. (B) CSF heparan sulfate declined rapidly after RGX-111 dosing and remained at or near normal throughout the duration of follow-up. Gray circles refer to CSF HS levels obtained from 29 control individuals without MPS I. (C) Subject's total urinary GAGs (orange) remained roughly constant throughout the study; though initially at upper limit of normal (dotted black line), it became elevated as the reference range declined with age. (D) Urinary heparan sulfate (uHS) and dermatan sulfate (uDS) levels were essentially unchanged throughout the study. Dotted red and blue horizontal lines indicate the upper limits of normal for uHS and uDS, respectively.

post dosing, and 0.19 nmol/h/mL (6.8% of control) at 276 weeks post dosing (Figure 1A). The subject's CSF HS was elevated at baseline (272 ng/mL; mean control CSF HS 69.1 ng/mL, range 140.5–22.0 ng/mL), declined to 137 ng/mL, which was within the normal range (−49.6% from baseline), at week 12, and remained consistently near normal through week 188, the last time point measured to date (Figure 1B). Pre-laronidase plasma IDUA levels were low at baseline and continued to be low throughout the study (data not shown). Urinary total GAGs were slightly elevated at baseline (29.58 mg/mmol creatinine; reference range 24 mg/mmol creatinine) and remained generally constant throughout the study (week 291, 28.82 mg/mmol creatinine, −19.2% from baseline, Figure 1C). Urinary HS (reference range <2.24 g/mol creatinine) and DS (reference range <4.18 g/mol creatinine) were elevated at baseline (37.31 and 20.54 g/mol creatinine, respectively). Although HS and DS were already reduced by more than 90% from diagnosis due to intravenous laronidase ERT, at week 291 both were further reduced to 13.78 and 14.01 g/mol creatinine, respectively (Figure 1D). While CSF IDUA enzyme levels declined after withdrawal of prednisone (week 12) and tacrolimus (week 24), CSF HS levels remained essentially unchanged.

Body fluid anti-IDUA serologies

Results of plasma and CSF anti-IDUA antibody titers are presented in Figure 2. Plasma anti-IDUA antibody was present at baseline (1:51,200 dilution) and declined to 1:6,400 post dosing and immunosuppression until week 24. As all immunosuppression was withdrawn by week 48, plasma anti-IDUA antibody returned to baseline at week 59 and reached a peak of 1:102,400 dilution at week 85. No infusion-associated reactions were observed with the increase in anti-IDUA titer, although this may have been associated with a slight increase in urinary HS and DS at week 87 that returned to baseline at week 113. Since week 178, the subject has retolerized to laronidase with subsequent antibody levels between 1:4,800 and 1:6,400 (Figure 2A). CSF anti-IDUA was present at baseline (1:80 dilution), declined to 1:40 post dosing and immunosuppression, and returned to 1:80 after removal of immunosuppression. At most recent measurement (week 276), CSF anti-IDUA titer was 1:20 (Figure 2B). Note that despite direct CNS dosing of RGX-111, the ratio of plasma to CSF anti-IDUA titers obtained at the same time ranged from 320:1 to 1,280:1.

Neurodevelopmental outcomes

At baseline, the patient was 20.7 months old with Bayley Scales of Infant and Toddler Development, 3rd Edition (BSID-III) values between 1 and 2 standard deviations below the normative mean, yielding a cognitive age equivalent (AEq) of 17 months (developmental quotient [DQ], 82.1), expressive communication AEq of 12 months (DQ 58.0), and fine motor AEq of 16 months (DQ 77.3). Ongoing skill acquisition at all post-RGX-111 dosing time points was observed. Cognitive, expressive communication, and fine motor AEqs increased at all assessments except for the 26 months post-dosing cognitive AEq, where reduced patient cooperation was documented. The 51-month post-dosing AEqs each exceeded the 42-month AEq BSID-III ceiling, requiring the usage of the Kaufman Assessment Battery for Children (K-ABC) at the 64-month post-dose final assessment, so this point could not be graphed. At a chronological age of 7 years, the subject's function on the cognitive, expressive communication, and fine motor subtests remained between 1 and 2 standard deviations from the normative

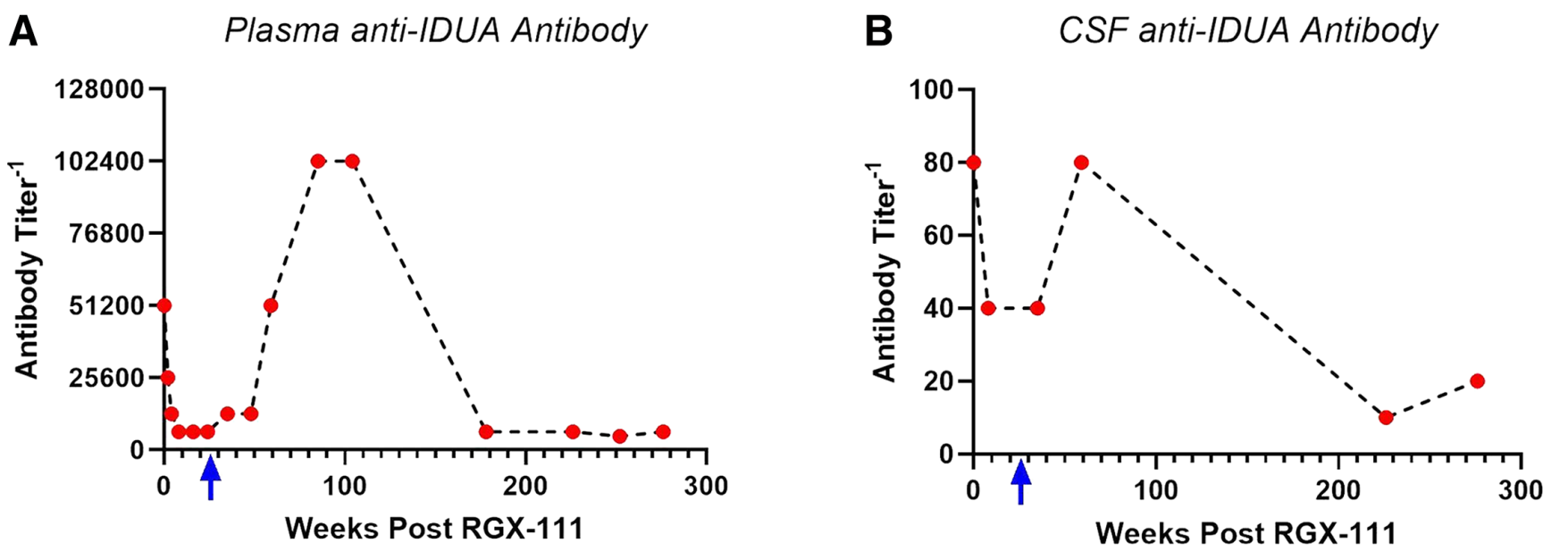

Figure 2. Measurement of plasma and CSF anti-IDUA antibody titers

(A) Plasma anti-IDUA antibodies declined with immunosuppression but surged to very high levels between year 1 and year 2 after removal of immunosuppression (blue arrow) before declining to post-RGX-111 levels at years 3, 4, and 5. (B) CSF anti-IDUA antibody titer was 1:80 at baseline and declined following administration of RGX-111 and immunosuppression; after withdrawal of immunosuppression (blue arrow), the titer returned to 1:80 and then decreased at years 4 and 5 to low levels.

mean (Figure 3). Clinically obtained audiology evaluations demonstrate normal hearing. He is bilingual in Arabic and English and is attending primary school in special education first grade with speech therapy (Video S1).

DISCUSSION

This report describes safety and clinical, biochemical, and neurodevelopmental outcomes 5.5 years following the first in-human intracisternal dosing of investigational human *IDUA* gene therapy (RGX-111; REGENXBIO) at 20.7 months of age in a male severe MPS I patient. Although intravenous laronidase was initiated upon diagnosis, its inability to cross the blood-brain barrier necessitated additional therapy to address neurocognitive sequelae of CNS IDUA deficiency and GAG accumulation. The investigational CNS gene therapy was chosen in favor of HSCT due to suboptimal stem cell HLA matching and HSCT-related fatalities in two MPS I-affected older siblings.

While MPS-related skeletal dysplasia increases the challenge of accessing the cisterna magna, a standardized protocol with thorough preprocedural MRI/MRA of the craniocervical junction allows full visualization of the anatomy, including the vertebrobasilar vasculature, to plan optimal strategy for intracisternal access. Our patient tolerated the procedure without complications and was discharged home the day after dosing. Most adverse events occurred in the first year after dosing and were mild. Only three adverse events—reduced activity level, reduced appetite, and vomiting—were considered possibly related to RGX-111 gene therapy, as the symptoms were suggestive of a viral syndrome and took place 1 week after dosing. One serious adverse event, acute otitis media, resulted from hospitalization owing to concern of mastoiditis; however, a CT scan did not identify mastoid fluid, and the child was discharged home with oral amoxicillin/clavulanate. Although no formal nerve conduction studies were performed, due to the protocol being a treatment investigational new drug (IND), the subject did not complain of paresthesias and did not demonstrate abnormal deep tendon reflexes, suggesting there was no clinically significant dorsal root ganglion (DRG) toxicity. This is very important, as toxicity to DRG sensory neurons was first noticed in animal model systems dosed with intravenous AAV[8]; our patient experience is in agreement with the neuropathology of nonhuman primates intracisternally dosed with AAV human *IDUA*, which did not identify any DRG lesions.[9] Finally, given the recent identification of an intraventricular CNS tumor demonstrating vector genome integration in a 5-year-old severe MPS I patient who had received ICM RGX-111 in the phase 1/2 open-label study (NCT03580083) at age 1 year,[10] the absence of brain or spine neoplasms in our child more than 5.5 years post RGX-111 is important to note. The child will continue to undergo annual brain and spine MRI surveillance for any CNS abnormality, especially neoplasm.

Rapid biochemical evidence of CNS transduction was evident, with measurable CSF IDUA enzyme activity and nearly 50% reduction in CSF HS observed 8 and 12 weeks, respectively, after treatment. These data also support that CSF GAG measurements may be an early biomarker that is reasonably likely to predict clinical benefit. Moreover, the child's continued developmental gains in the 5 years after dosing suggests potential efficacy of the investigational intra-CNS *IDUA* gene therapy. The child's most recent cognitive AEq of 62 months exceeds the highest cognitive AEq achieved (25 months) in a study of 32 untransplanted severe MPS I patients, whose AEq trajectories inflected toward regression at 50 months.[11] Enrollment in school and initiation of developmental therapies is also expected to continue improving his cognitive function, expressive communication, and motor skills. Although the subject's most recent CSF enzyme activity declined to 6.8% of control, his CSF HS did not increase appreciably. This is consistent with the abundant clinical experience with allogeneic HSCT for treatment of severe MPS I, where small amounts of CSF IDUA appear sufficient to reduce HS levels and preserve neurocognition.[12,13] The biochemical and neurocognitive outcomes of this first-in-human dosing of RGX-111 indicate that intracisternal gene therapy for severe MPS I averts the severe neurodegenerative natural history of the condition without the significant risks of immunocompromise, engraftment syndrome, donor graft failure with autologous recovery, and death presented by the current standard of care, which is allogeneic HSCT.

A limitation of the investigational intracisternal gene therapy in this child is the need to continue intravenous ERT for somatic MPS I disease. We did not observe any appreciable changes in plasma IDUA activity, urinary GAGs, HS, or DS after RGX-111 dosing. This is clinically consistent with the development of bilateral

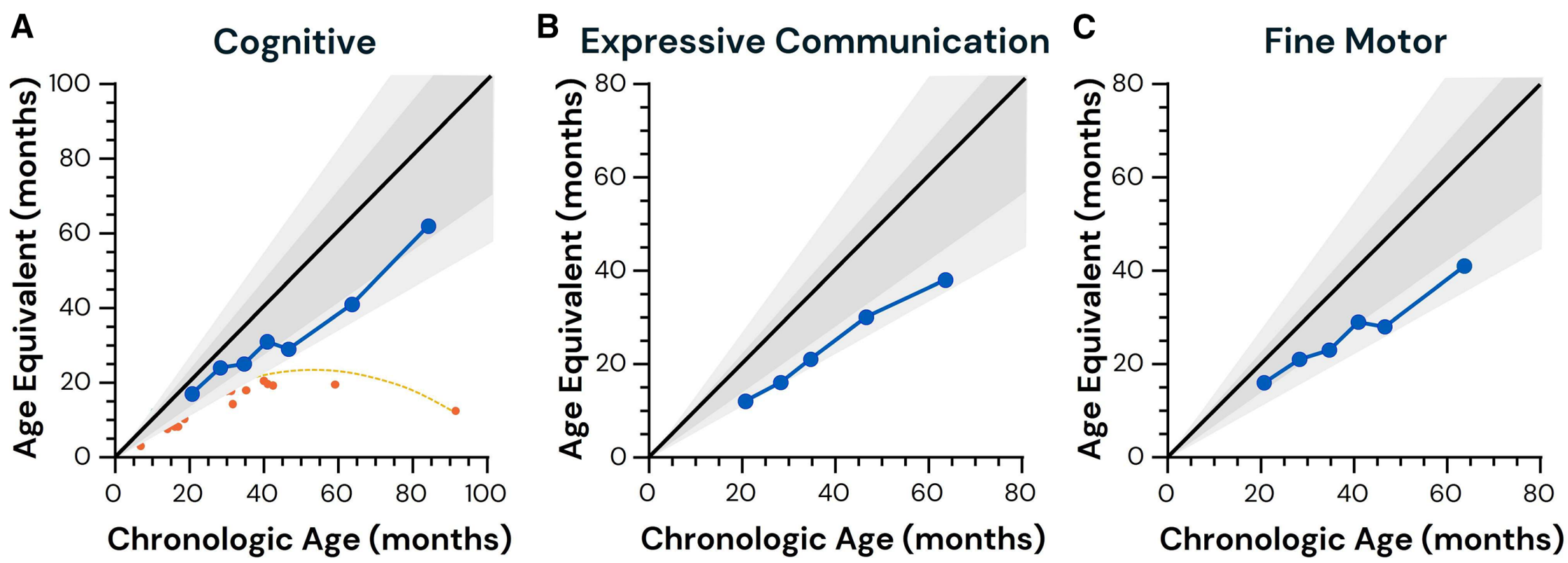

Figure 3. Longitudinal neurodevelopmental age-equivalent scores

Longitudinal neurodevelopmental age-equivalent scores (blue circles) for the subject's (A) cognition, (B) expressive communication, and (C) fine motor function increased with age, indicating ongoing acquisition of developmental milestones without regression. Scores remain between 1 and 2 standard deviations below the mean for age. Data at 72 months chronological age (+52 months post dosing) was not plotted because subject tested above the BSID-III ceiling of 42 months for each category; only cognitive age equivalent is plotted at 84 months chronological age (+64 months post dosing), as the K-ABC does not have a corresponding category for expressive communication and fine motor function. Note that untreated severe MPS I cognitive age equivalents (orange) plateau at an age equivalent of 24 months at a chronological age of 50 months, then progressively decline. Black line indicates the mean (age equivalent = chronological age); the light-gray areas indicate age equivalents ±1 standard deviation, and the dark-gray areas indicate age equivalents ±2 standard deviations from mean.

corneal clouding, cervical spinal stenosis, thoracolumbar kyphosis, joint contractures, and genu valgum in the subject; ophthalmologic and orthopedic symptoms of MPS I are well described to persist despite intravenous ERT.[14,15] The development of MPS I orthopedic manifestations in the subject is in contrast to the outcomes of eight severe MPS I children treated with lentiviral IDUA autologous hematopoietic stem progenitor cell transplantation (NCT03488394), who expressed supranormal blood IDUA enzyme levels and demonstrated improvement of shoulder and knee joint mobility in conjunction with expression of CSF IDUA, reduction of CSF HS, and progressive acquisition of cognitive skills.[16–19]

While these data are from a single patient, they suggest that RGX-111 gene therapy delivered into the CNS to treat MPS I generates IDUA expression and GAG clearance within the brain, which are associated with sustained biochemical effects and cognitive benefits, more rapidly than conventional allogeneic HSCT, which requires time for stem cells to engraft, migrate to the brain, and secrete IDUA for cross-correction and catabolism of GAGs. In conjunction with identification of presymptomatic Hurler syndrome infants with universal newborn screening, investigational gene therapy has the potential to allow earlier neurologic benefit as typically HSCT-treated patients experience neurocognitive decline for up to a year before stabilizing and increasing.[2] Universal newborn screening for MPS I has enabled early oligosymptomatic diagnosis and initiation of CNS-directed therapies to likely further enhance neurocognitive outcomes. HSCT-related adverse effects, including hepatic veno-occlusive disease, thrombotic microangiopathy, donor graft failure with autologous recovery, and death could potentially be avoided. Long-term clinical, biochemical, and neurocognitive follow-up is in progress to document the trajectory of CSF IDUA, HS, neurodevelopment, and other markers in this subject in addition to others who have received intracisternal RGX-111 as participants in the phase 1/2 open-label study.

MATERIALS AND METHODS

Human subjects

Following Food and Drug Administration approval of a single-patient IND application for an open-label, single-dose, ICM administration of RGX-111 (#19145), and provision of parental informed consent (see supplemental text: informed consent, CHOC IRB #190671), screening procedures were initiated at 20.3 months chronological age. Parents provided consent for videography and photography as well as sharing of video images for publication purposes. Immunosuppression (for full regimen, see Supplemental Text, Protocol) was continued for a total of 48 weeks after RGX-111 dosing. To summarize, sirolimus 1 mg/m^2/dose $\times$ 3 doses every 4 h was given orally 2 days prior to RGX-111; 0.25 mg/m^2/dose was given orally twice a day for the next 48 weeks, which achieved the target trough blood levels of 1–3 ng/mL. Methylprednisolone 10 mg/kg was administered one time, intravenously, 30 min prior to ICM RGX-111. Prednisone 0.5 mg/kg/dose was given orally once daily beginning the day after RGX-111 for 2 weeks, reduced to 0.35 mg/kg/dose once daily for 2 weeks, further reduced to 0.2 mg/kg/dose once daily for 2 weeks, and finally 0.1 mg/kg/dose once daily for 2 weeks. Tacrolimus 0.05 mg/kg/dose given orally twice daily was initiated the day after RGX-111 for 24 weeks, which was sufficient to maintain trough blood levels of 2–4 ng/mL, then reduced by 12.5% per week until discontinuation at 32 weeks. Follow-up assessments have been performed periodically for more than 5.5 years after dosing. At each follow-up visit, biomarker laboratory assessments were performed prior to administration of intravenous laronidase ERT.

Biochemical quantifications

IDUA enzyme activity was assessed using 10 μL of CSF or plasma incubated with 4-methylumbelliferyl α-L-iduronide substrate (final concentration 0.25 mM, Biosynth) in formate buffer (pH 3.5) for 1 h at 37°C in a 96-well plate. Reactions were quenched with 180 μL of glycine carbonate buffer (pH 10.5), and fluorescence measurements were obtained using an Infinite M Plex spectrofluorophotometer (Tecan) at excitation and emission wavelengths of 360 nm and 450 nm, respectively. Bovine serum albumin was utilized as negative control for the IDUA reaction. Control CSF IDUA was measured from 17 pediatric subjects with mitochondrial disorders (CHOC IRB #130990). Measurements were made in triplicate and blinded to subject identity and treatment administered. Substrate background was determined using 10 μL of formate buffer instead of the biological sample; the limit of detection was defined as any signal exceeding 10% of background and was identified to be 0.15 nmol/h/mL.

CSF HS was determined using a proprietary liquid chromatography-tandem mass spectrometry (LC-MS/MS) method developed at CAP-accredited and CLIA-certified Frontage Laboratories (Exton, PA). In brief, HS polysaccharides were digested by heparanases into disaccharides, which were then derivatized before being analyzed with LC-MS/MS; quantitation was performed using calibration curves generated from isotope-labeled internal standard. Control CSF HS was measured in 29 samples obtained from unaffected individuals. Quantification of total urinary GAGs was performed at CAP-accredited and CLIA-certified Greenwood Genetic Center Biochemical Genetics Laboratory (Greenwood, SC) using 1,9-dimethyl-methylene blue incorporation and spectrophotometry, and results were normalized to urine creatinine.

Neurodevelopmental assessments

Neurodevelopmental outcomes were assessed at baseline and annually post RGX-111 dosing by one rater (M.T.) initially using BSID-III. BSID-III AEq data are presented from the cognitive, expressive communication, and fine motor subtests. The AEq score represents the mean age at which the subtest raw score is obtained in the normative sample. Patient AEq is presented by chronological age in the figures together with standard deviations from the normative mean. Normative data and standard deviations of AEqs were derived from standard scores from the BSID-II manual.[20] When the subject's AEq scores exceeded 48 months, the ceiling age for the BSID-III, the K-ABC was utilized.

DATA AND CODE AVAILABILITY

De-identified participant data and images collected for the study will be made available for review upon reasonable request to the corresponding author with a signed data access agreement. The study protocol and informed consent are available as supplemental information.

ACKNOWLEDGMENTS

The investigational RGX-111 product was provided by REGENXBIO. The study was partially supported by REGENXBIO. R.Y.W. and S.-H.K. receive support from the Campbell Foundation of Caring.

AUTHOR CONTRIBUTIONS

R.Y.W., conceptualization, methodology, supervision, project administration, investigation, data curation, visualization, writing – original draft, and writing – review & editing; N.M., project administration, investigation, data curation, and writing – review & editing; S.-H.K., resources, investigation, validation, visualization, and writing – review & editing; T.B., investigation and writing – review & editing; M.T., investigation and writing – review & editing; R.C.C., investigation and writing – review & editing; D.P., methodology, resources, and writing – review & editing; J.B., methodology, funding acquisition, and writing – review & editing; M.G., methodology, resources, and writing – review & editing; Y.C., methodology and writing – review & editing; P.F., supervision, resources, funding acquisition, and writing – review & editing; L.P., supervision, resources, and writing – review & editing.

DECLARATION OF INTERESTS

D.P., J.B., M.G., Y.C., P.F., and L.P. are employees of REGENXBIO, Inc.

SUPPLEMENTAL INFORMATION

Supplemental information can be found online at https://doi.org/10.1016/j.ymthe.2026.04.016.

REFERENCES

1. Neufeld, E.F., and Muenzer, J. (2019). The Mucopolysaccharidoses. In The Online Metabolic and Molecular Bases of Inherited Disease, D. Valle, S. Antonarakis, A. Ballabio, A. Beaudet, and G.A. Mitchell, eds. (New York, NY: McGraw-Hill Education).
2. Shapiro, E.G., Nestrasil, I., Rudser, K., Delaney, K., Kovac, V., Ahmed, A., Yund, B., Orchard, P.J., Eisengart, J., Niklason, G.R., et al. (2015). Neurocognition across the spectrum of mucopolysaccharidosis type I: Age, severity, and treatment. Mol. Genet. Metab. *116*, 61–68.
3. Archer, L.D., Langford-Smith, K.J., Bigger, B.W., and Fildes, J.E. (2014). Mucopolysaccharide diseases: a complex interplay between neuroinflammation, microglial activation and adaptive immunity. J. Inherit. Metab. Dis. *37*, 1–12.
4. Clarke, L.A., Wraith, J.E., Beck, M., Kolodny, E.H., Pastores, G.M., Muenzer, J., Rapoport, D.M., Berger, K.I., Sidman, M., Kakkis, E.D., et al. (2009). Long-term efficacy and safety of laronidase in the treatment of mucopolysaccharidosis I. Pediatrics *123*, 229–240.
5. Eisengart, J.B., Rudser, K.D., Xue, Y., Orchard, P., Miller, W., Lund, T., Van der Ploeg, A., Mercer, J., Jones, S., Mengel, K.E., et al. (2018). Long-term outcomes of systemic therapies for Hurler syndrome: an international multicenter comparison. Genet. Med. *20*, 1423–1429.
6. Aldenhoven, M., Wynn, R.F., Orchard, P.J., O'Meara, A., Veys, P., Fischer, A., Valayannopoulos, V., Neven, B., Rovelli, A., Prasad, V.K., et al. (2015). Long-term outcome of Hurler syndrome patients after hematopoietic cell transplantation: an international multicenter study. Blood *125*, 2164–2172.
7. Aldenhoven, M., Jones, S.A., Bonney, D., Borrill, R.E., Coussons, M., Mercer, J., Bierings, M.B., Versluys, B., van Hasselt, P.M., Wijburg, F.A., et al. (2015). Hematopoietic cell transplantation for mucopolysaccharidosis patients is safe and effective: results after implementation of international guidelines. Biol. Blood Marrow Transpl. *21*, 1106–1109.
8. Hinderer, C., Katz, N., Buza, E.L., Dyer, C., Goode, T., Bell, P., Richman, L.K., and Wilson, J.M. (2018). Severe Toxicity in Nonhuman Primates and Piglets Following High-Dose Intravenous Administration of an Adeno-Associated Virus Vector Expressing Human SMN. Hum. Gene Ther. *29*, 285–298.
9. Hordeaux, J., Hinderer, C., Buza, E.L., Louboutin, J.P., Jahan, T., Bell, P., Chichester, J.A., Tarantal, A.F., and Wilson, J.M. (2019). Safe and Sustained Expression of Human Iduronidase After Intrathecal Administration of Adeno-Associated Virus Serotype 9 in Infant Rhesus Monkeys. Hum. Gene Ther. *30*, 957–966.
10. REGENXBIO (2026). REGENXBIO Announces Regulatory Update on Ultra Rare MPS Programs (REGENXBIO Press Releases).
11. Shapiro, E.G., Whitley, C.B., and Eisengart, J.B. (2018). Beneath the floor: re-analysis of neurodevelopmental outcomes in untreated Hurler syndrome. Orphanet J. Rare Dis. *13*, 76.

12. Shull, R.M., Hastings, N.E., Selcer, R.R., Jones, J.B., Smith, J.R., Cullen, W.C., and Constantopoulos, G. (1987). Bone marrow transplantation in canine mucopolysaccharidosis I. Effects within the central nervous system. J. Clin. Invest. *79*, 435–443.

13. Visigalli, I., Delai, S., Politi, L.S., Di Domenico, C., Cerri, F., Mrak, E., D'Isa, R., Ungaro, D., Stok, M., Sanvito, F., et al. (2010). Gene therapy augments the efficacy of hematopoietic cell transplantation and fully corrects mucopolysaccharidosis type I phenotype in the mouse model. Blood *116*, 5130–5139.

14. Hampe, C.S., Wesley, J., Lund, T.C., Orchard, P.J., Polgreen, L.E., Eisengart, J.B., McLoon, L.K., Cureoglu, S., Schachern, P., and McIvor, R.S. (2021). Mucopolysaccharidosis Type I: Current Treatments, Limitations, and Prospects for Improvement. Biomolecules *11*, 189.

15. Miller, B.S., Fung, E.B., White, K.K., Lund, T.C., Harmatz, P., Orchard, P.J., Whitley, C.B., and Polgreen, L.E. (2023). Persistent bone and joint disease despite current treatments for mucopolysaccharidosis types I, II, and VI: Data from a 10-year prospective study. J. Inherit. Metab. Dis. *46*, 695–704.

16. Gentner, B., Tucci, F., Galimberti, S., Fumagalli, F., De Pellegrin, M., Silvani, P., Camesasca, C., Pontesilli, S., Darin, S., Ciotti, F., et al. (2021). Hematopoietic Stem- and Progenitor-Cell Gene Therapy for Hurler Syndrome. N. Engl. J. Med. *385*, 1929–1940.

17. Consiglieri, G., Tucci, F., De Pellegrin, M., Guerrini, B., Cattoni, A., Risca, G., Scarparo, S., Sarzana, M., Pontesilli, S., Mellone, R., et al. (2024). Early skeletal outcomes after hematopoietic stem and progenitor cell gene therapy for Hurler syndrome. Sci. Transl. Med. *16*, eadi8214.

18. Tucci, F., Uria Oficialdegui, M.L., Consiglieri, G., Cossutta, M., Filisetti, C., Fumagalli, F., Butera, C., Santangelo, R., Colombo, M., Manitto, M.P., et al. (2025). Non-neurological, non-skeletal outcomes after hematopoietic stem and progenitor cell-gene therapy (OTL-203) for Hurler syndrome. Mol. Ther. *34*, 443–454.

19. Tucci, F., Consiglieri, G., Cossutta, M., and Bernardo, M.E. (2025). From trial to transformation: insights into hematopoietic stem cell-gene therapy for Hurler syndrome. Expert Opin. Biol. Ther. *25*, 1271–1274.

20. Cho, Y., Forsberg, M., and Phillips, D. (2021). Clinical and numerical presentation of neurocognitive assessments for MPS II patients using the Bayley Scales of Infant Development- version 3 (BSID-III). Mol. Genet. Metab. *132*, S25.

YMTHE, Volume *34*

Supplemental Information

First-in-human intracisternal dosing of RGX-111 in severe MPS I is well tolerated and generates sustained neurodevelopment without HSCT

Raymond Y. Wang, Nina Movsesyan, Shih-Hsin Kan, Tammam Beydoun, Mery Taylor, Richard C. Chang, Dawn Phillips, Jenna Burke, Michelle Gilmor, Yoonjin Cho, Paulo Falabella, and Laura Pisani

Supplemental Text: Protocol

Physician Initiated, Single Center, Single Patient Administration of Intracisternal RGX-111 Gene Therapy for Severe Mucopolysaccharidosis Type I

Protocol Date: 11 July 2019

Sponsor / Principal Investigator: Raymond Wang, M.D.

Sub Investigators: Richard Chang, M.D., Tammam Beydoun, D.O.

Drug: RGX-111 (AAV9.CB7.hIDUA, recombinant adeno-associated virus serotype 9 capsid containing human α-L-iduronidase expression cassette)

Treatment Site: CHOC Children's Hospital, Orange, California

Contact Information:

Name: Raymond Y. Wang, M.D.
Tel: (714) 509-8443
Fax: (714) 509-4531
Email: rawang@choc.org

Study Medication Provider: REGENXBIO, Inc

IND Number: 19145

CHOC IRB Number: 190671

Rationale for Intended Use of Drug
The subject is a 16 month old, 12 kg male child with severe MPS I (*IDUA* homozygous p.R628*/R628*, known pathogenic mutation). Family history: 3 older affected siblings with identical condition. Oldest sibling diagnosed too late for hematopoietic stem cell transplantation (HSCT), which is the standard of care for severe MPS I, and is currently 18 years old with severe disease manifestations including: nonambulatory, nonverbal with severe hydrocephalus, corneal clouding / severely reduced visual acuity, airway obstruction, tracheostomy/gastrostomy dependent.

Subject's second and third oldest siblings underwent HSCT but their non-Caucasian ethnicity made for poor marrow/cord blood stem cell matches and best options were 4 of 6 HLA match umbilical cord lots. Both siblings died from transplant-related complications due to suboptimal matching; subject would suffer a likely outcome of death as recent assessments of available stem cell lots do not offer any better matched candidates.

There are no other standard of care therapies that can preserve neurocognitive function in severe MPS I. Enzyme replacement therapy (ERT) with Aldurazyme® (laronidase, recombinant human alpha iduronidase enzyme, Biomarin Pharmaceuticals, Novato, CA) does not cross the blood brain barrier and cannot treat the neurocognitive regression that occurs in severe MPS I.

Nonclinical studies have shown that RGX-111, an adeno-associated virus (AAV)-mediated gene transfer of IDUA, when delivered into the central nervous system (CNS), can increase IDUA protein expression in a robust and rapid manner, resulting in rapid and near-complete correction of biochemical changes resulting from IDUA deficiency. (Hinderer 2016). Evidence of positive systemic effects were observed as well. Treatment with RGX-111 offers a potential therapeutic approach to address the CNS manifestations of MPS I. Published literature indicates intervention with stem cell transplant must occur ideally prior to 16 months for preservation of neurocognitive function, so for best neurocognitive outcomes this study proposes a one-time, intracisternal (IC) administration of 1e10 genome copies (GC) per gram of brain mass of the drug RGX-111 (AAV9.CB7.hIDUA, recombinant adeno-associated virus serotype 9 capsid containing human α-L-iduronidase expression cassette), as soon as possible, with the possibility that RGX-111 will transduce cells of CNS and result in endogenous synthesis of human iduronidase in the CNS to mitigate neurocognitive deterioration and preserve neurocognitive development.

Subject is not eligible for the current RGX-111 study (NCT03580083) as it mandates subjects be 6 years or older. For this reason, this single patient IND request is being made on behalf of subject.

Description of Severe Mucopolysaccharidosis Type I
Mucopolysaccharidosis type I (MPS I) is a recessively inherited genetic lysosomal storage disease caused by a deficiency of α-L-iduronidase (IDUA) leading to an accumulation of glycosaminoglycans (GAGs) in tissues of affected patients. Since GAGs build up in the CNS and the rest of the body, MPS I patients suffer diverse disease sequelae, including neurocognitive deficits. Severe MPS I (Hurler syndrome) typically involves significant developmental delay and cognitive decline. In untreated Hurler syndrome, symptoms emerge shortly after birth and progress rapidly, such that most patients with Hurler syndrome exhibit severe cognitive decline at 2 – 3 years of age after an initial period of normal development and die within the 1st decade of life.

Hematopoietic stem cell transplantation is the standard of care for Hurler syndrome in the United States, demonstrating in multiple cohort studies and meta-analyses improvements in survival, growth, cardiac and respiratory function, mobility, hearing, visual acuity, and CNS manifestations such as neurocognition. The rationale for treatment is known as 'cross correction,' in which the subject's own cells internalize α-L-iduronidase that is secreted from grafted stem cells which have taken residence within the subject. The internalized enzyme then breaks down the GAGs and mitigates storage-related symptoms.

While intravenous Aldurazyme® ERT is established standard of care for somatic manifestations of MPS I, the drug does not cross the blood-brain barrier and therefore cannot treat the neurocognitive sequelae of severe MPS I. In contrast, HSCT is able to treat neurocognitive sequelae because a subpopulation of donor stem cells

migrates across the blood-brain barrier, differentiates into the immune cells of the CNS, and secretes α-L-iduronidase for the recipient's CNS cells to uptake and cross-correct.

Subject's Medical History and Previous Treatments

Due prior family history, prenatal diagnosis for MPS I was offered to parents, who declined. He was brought for first evaluation at 2 months of age and large bilateral inguinal hernias were identified. Biochemical and molecular testing subsequently confirmed severe MPS I disease, with elevated urinary total GAGs, elevated heparan and dermatan sulfate GAGs, deficient α-L-iduronidase enzyme (1.14 nmol/hr/mg prot, reference range 6 – 71.4), and homozygous *IDUA* p.R628* mutations. Diagnostic evaluations identified mild aortic root dilatation (Z score +2.5), hepatomegaly (11.3 cm), and dysostosis multiplex.

Subject was evaluated for HSCT; the best match was a 4 of 6 umbilical cord unit. HSCT was offered for treatment for subject, but given the fatal outcomes in two prior MPS I affected children – which were due to severe engraftment / graft-versus-host complications – the family declined HSCT.

He began intravenous Aldurazyme® ERT at a standard dose of 0.58 mg/kg/week at 6 months of age and has continued for the past 11 months without infusion-associated reactions or adverse events. He has undergone successful bilateral inguinal herniorrhaphy. Developmentally, he is walking unsupported, is able to feed himself, and has three words. No developmental regression has been noted yet. Objective evaluations at 16 months of age demonstrate normal growth (length 20th percentile, weight 90th percentile, head circumference 94th percentile); mild coarse facial features, minimal corneal clouding, and hip, knee, and elbow joint contractures with a lumbosacral gibbus and brachydactyly. The relative macrocephaly and physical examination findings are consistent with stigmata of MPS I that current Aldurazyme® treatment is unable to fully mitigate. He shall continue on intravenous Aldurazyme® throughout this treatment protocol.

Proposed Method of Administration of Drug, Dose, and Duration of Therapy

Method of administration of RGX-111: computerized tomography-guided intra-cisterna magna injection

Dosage: 1 x 10^{10} genome copies per gram of patient brain mass (estimated via quantitation of brain magnetic resonance imaging), diluted 1:1 (v/v) with Elliotts B artificial CSF solution.

Duration of therapy: One time administration

Adjunctive (immune suppression) therapy:

Methylprednisolone, 10 mg/kg intravenously, one time prior to RGX-111 administration.

Prednisone, 0.5 mg/kg/day orally, once daily starting day following RGX-111 administration and gradually tapering to discontinuation by +12 weeks following RGX-111 administration.

Tacrolimus, 0.05 mg/kg/dose orally, twice daily starting day following RGX-111 administration with dose titration to achieve target blood level of 2 – 4 ng/mL, x 24 weeks. Dose shall be tapered over 8 weeks to discontinuation by +32 weeks following RGX-111 administration.

Sirolimus, 1 mg/m^2 every 4 hours x 3 doses, 2 days prior to RGX-111 administration, then 0.25 mg/m^2/dose orally, twice daily with dose titration to achieve target blood level of 1 – 3 ng/mL, x 48 weeks.

Prophylactic therapy:

Trimethoprim/sulfamethoxazole, 5 mg/kg orally, Monday / Wednesday / Friday once each dosing day. For prophylaxis against *Pneumocystis carinii* infection

Antifungal prophylaxis against *Candida* and other fungal infections is to be initiated if the ANC is < 500 mm3. The treatment regimen will be determined through local site standard of care.

Description of Clinical Procedures, Laboratory Tests, and Monitoring

Clinical Procedures

Following informed consent provided by parents, baseline assessments would be performed. These assessments can occur up to three weeks prior to RGX-111 dosing to allow for scheduling of all requisite procedures.

Baseline assessments include: medical history and physical examination; height / weight / head circumference; abdominal ultrasound to quantify liver and spleen size; and electrocardiogram.

Neurocognitive testing (Bayley Scales of Infant Development III) will occur at baseline, 24, 48, 78 and 104 weeks.

RGX-111 will be administered via CT guided intra-cisternal injection. The approach shall be from the posterior aspect of the neck, directed towards the foramen of Magendie. CNS anatomy has been reviewed in detail via magnetic resonance imaging. No anatomic anomalies or contraindications to this route have been identified.

Physical and neurologic examination will occur immediately following, and one day after RGX-111 dosing.

Subsequently, these examinations and vital signs will occur at 8, 24, 56, 78, and 104 weeks post-RGX-111 dosing.

Lumbar puncture for assessment of CSF opening pressure and obtaining spinal fluid for laboratory testing will take place at baseline and 8, 24, and 56 weeks after RGX-111 administration.

Subject shall continue to receive weekly, standard-of-care Aldurazyme® ERT throughout this protocol. The option to potentially discontinue intravenous ERT will be based on the clinical judgement of the PI; additional information that may be useful for the decision to stop ERT are trough measurements (based on ERT dosing) of whole blood IDUA activity and urine GAGs up to the Week 24 visit and liver volume by ultrasound. The Week 32, 40, and 48 visits will include additional monitoring of the subject's plasma IDUA activity and urine GAGs levels. IV ERT will be restarted if any of following criteria are met should subject be off Aldurazyme® ERT: increase in urinary GAGs levels 2 times above the level measured at the week 24 visit, or any change in other safety parameters to warrant a restart of IV ERT or as deemed necessary by the PI.

Follow-up abdominal ultrasounds will occur at 24, 56, 78, and 104 weeks post-RGX-111 dosing.
Echocardiogram and electrocardiograms will occur at 56 and 104 weeks post-RGX-111 dosing.
Brain magnetic resonance imaging will be performed at 56 and 104 weeks post-RGX-111 dosing.

Laboratory Tests

Complete blood count (CBC), comprehensive metabolic panel (CMP), lipid panel, coagulation panel, and urinalysis will be obtained before first sirolimus dosing, which is two days prior to administration of RGX-111.

Tacrolimus and sirolimus blood levels for drug titration purposes will be obtained 1, 2, 3, 4, 8, 16, and 24 weeks following RGX-111 administration. Since tacrolimus will be discontinued by week 32, only sirolimus levels will be obtained at 32 and 40 weeks post-RGX-111 administration.

CBC, CMP, and urinalysis will be obtained the day following administration of RGX-111; then 2, 4, 8, 16, 24, 32, 40, 48, 56, 78, and 104 weeks afterwards.

Blood (α-L-iduronidase enzyme activity and GAGs) and urine biomarkers (GAGs) will be obtained at baseline and at 2, 4, 8, 16, 24, 32, 40, 48, 56, 78, and 104 weeks (ideally coinciding with pre-Aldurazyme® ERT for trough α-L-iduronidase quantification) following RGX-111 administration.

Serum and urine RGX-111 quantitative PCR will be obtained at baseline and at 1, 4, 8, 16, and 24 weeks post-RGX-111 dosing.

Serum immune response monitoring (anti-AAV9 and anti-α-L-iduronidase antibody quantification) will be obtained one day prior to, then 2, 8, 16, 24, 32, 56, 78, and 104 weeks post-RGX-111 administration.

ELISPOT assay (for subject's immune response to AAV9 vector and α-L-iduronidase enzyme) will be performed at baseline and at 8, 24, 48, 56, 78, and 104 weeks following RGX-111 administration.

T_{reg} assay will be performed at baseline and at 24, 56, 78, and 104 weeks following RGX-111 administration.

Cerebrospinal fluid testing at baseline, week 8, 24, and 56 will include: protein, glucose, cell count, RGX-111 quantitative PCR, immune response, and biomarker quantification (α-L-iduronidase enzyme activity and GAGs).

Monitoring
Subject will be hospitalized the day prior to, the day of IC RGX-111, and discharged the day after IC RGX-111 if no adverse events are noted (normal activity level, movement, gait, and strength of extremities).

Concomitant medications shall be collected every 4 weeks.

Adverse event / serious adverse event (as defined by CTCAE version 5.0) reporting shall take place throughout the 104 week study period.

Appendix: Information Regarding Devices Utilized to Deliver RGX-111 via IC Injection

Ancillary Components included in the "Pharmacy Kit for Intrathecal / Intracisternal Administration (containing commercial ambient diluent)"

Component	Vendor	Part Number	Quantity Included	Description of Component Use	Regulatory Status
1 mL syringe	Becton Dickinson (BD™)	309628	2	Withdrawal of IP and diluent and prepared dose	K110771[a]
3 mL syringe	Becton Dickinson (BD™)	309657	2		
5 mL syringe	Becton Dickinson (BD™)	309646	2		
10 mL syringe	Becton Dickinson (BD™)	302995	2		
18G Blunt Fill Needle with Filter	Becton Dickinson (BD™)	305211	1	Withdrawal of Elliotts B® Solution	NA[b]
18G Blunt Fill Needle	Becton Dickinson (BD™)	305180	2	Transfer of withdrawn IP and diluent into dosing syringe	
Alcohol Prep Pads	Uline	S-16183	10	IP vial	NA
Vial Adapters	West Pharmaceuticals	8070117	10	Withdrawal of IP from vials	K001293 [a]
Elliotts B® Solution	Lukare Medical	28002	1	Diluent	NDC<br>55792-007-10
Tip Cap	B Braun	R2000	1	Closure of primed dosing syringe	NDC/WSN 08021-6544-96
Sterile Label / Pen	Various	Various	1	Labeling of syringes	NA
Sterility Maintenance Bag	Various	3312ST	1	Storage of prepared dosing syringe	NA

[a]510(k) number

[b]FDA Class 1 exempt since classified as a manual surgical instrument for general use, meets ISO guidelines for needles (7864 and 9626)

Ancillary Components included in the “Surgery Kit for Intrathecal / Intracisternal Administration”

Component	Vendor	Part Number	Quantity Included	Description of Component Use	Regulatory Status
25 G × 2” Short Spinal Needle with Quincke Bevel	Becton Dickinson (BD™)	405078	2	Administration of IP into patient	D018795[a]
25 G × 3” Short Spinal Needle with Quincke Bevel	Becton Dickinson (BD™)	405170	2		
25 G × 4 11/16” Spinal Needle with Quincke Bevel	Becton Dickinson (BD™)	405234	2		
22 G x 5” Spinal Needle with Quincke Bevel	Becton Dickinson (BD™)	405148	2		
20 G x 3” Spinal Needle with Quincke Bevel	Becton Dickinson (BD™)	405172	1	Optional introducer needle for stabilizing 25G spinal needle	
18 G x 3” Spinal Needle with Quincke Bevel	Becton Dickinson (BD™)	405174	1	Optional introducer needle for stabilizing 22G spinal needle	
T-connector extension set with female luer lock adapter, approximate length 6.7”	Baxter	2N3339	1	Administration of IP into patient	K921899[b]
